# Socially Mediated Shift in Neural Circuits Activation Regulated by Synergistic Neuromodulatory Signaling

**DOI:** 10.1523/ENEURO.0311-23.2023

**Published:** 2023-11-27

**Authors:** Katie N. Clements, Sungwoo Ahn, Choongseok Park, Faith K. Heagy, Thomas H. Miller, Miki Kassai, Fadi A. Issa

**Affiliations:** 1 Department of Biology, East Carolina University, Greenville, NC 27858; 2 Department of Mathematics, East Carolina University, Greenville, NC 27858; 3 Department of Biochemistry and Molecular Biology, East Carolina University, Greenville, NC 27858; 4 Department of Mathematics, North Carolina A&T State University, Greensboro, NC 27411

**Keywords:** aggression, neural plasticity, neuromodulation, sensory motor integration, zebrafish

## Abstract

Animals exhibit context-dependent behavioral decisions that are mediated by specific motor circuits. In social species these decisions are often influenced by social status. Although social status-dependent neural plasticity of motor circuits has been investigated in vertebrates, little is known of how cellular plasticity translates into differences in motor activity. Here, we used zebrafish (*Danio rerio*) as a model organism to examine how social dominance influences the activation of swimming and the Mauthner-mediated startle escape behaviors. We show that the status-dependent shift in behavior patterns whereby dominants increase swimming and reduce sensitivity of startle escape while subordinates reduce their swimming and increase startle sensitivity is regulated by the synergistic interactions of dopaminergic, glycinergic, and GABAergic inputs to shift the balance of activation of the underlying motor circuits. This shift is driven by socially induced differences in expression of dopaminergic receptor type 1b (Drd1b) on glycinergic neurons and dopamine (DA) reuptake transporter (DAT). Second, we show that GABAergic input onto glycinergic neurons is strengthened in subordinates compared with dominants. Complementary neurocomputational modeling of the empirical results show that drd1b functions as molecular regulator to facilitate the shift between excitatory and inhibitory pathways. The results illustrate how reconfiguration in network dynamics serves as an adaptive strategy to cope with changes in social environment and are likely conserved and applicable to other social species.

## Visual Abstract

**Figure:**
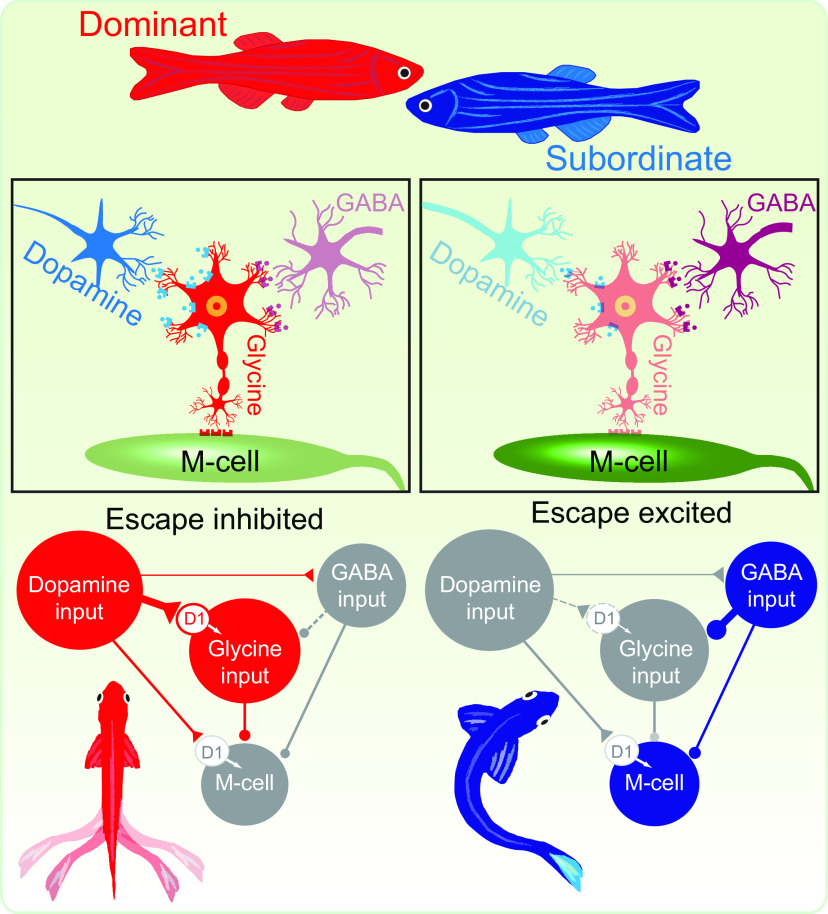

## Significance Statement

The neural mechanisms underlying social behavior remain poorly understood. The study shows how synaptic plasticity of dopaminergic, GABAergic, and glycinergic inputs synergistically modulate zebrafish startle escape and swim behavior in a social status-dependent manner. Socially driven changes in *drd1b* expression in glycinergic neurons shifts the balance between excitatory (dopaminergic) and inhibitory (glycinergic and GABAergic) pathways highlighting the importance of *drd1b* as a molecular regulator of adaptive social behavior.

## Introduction

Social status-dependent modulation of neural circuits has been extensively investigated in vertebrate and invertebrate systems (Edwards and Kravitz, 1997; Whitaker et al., 2011). Social factors can shift the balance among behaviorally relevant outputs by modulating the excitability of individual underlying neural networks. However, the effects of social status on the interplay between multiple neuromodulatory networks responsible for balancing the excitation of behaviorally relevant outputs remains poorly characterized. Using zebrafish, we investigated how social dominance influences the switch in network activity between two spinal motor circuits: startle escape and swim; focusing on the dynamic interplay between multiple neuromodulatory networks. We show that social dominance regulates the synergistic interaction of dopaminergic, glycinergic, and GABAergic inputs to control spinal motor circuit activity in a social status-dependent manner.

**Figure 1.**
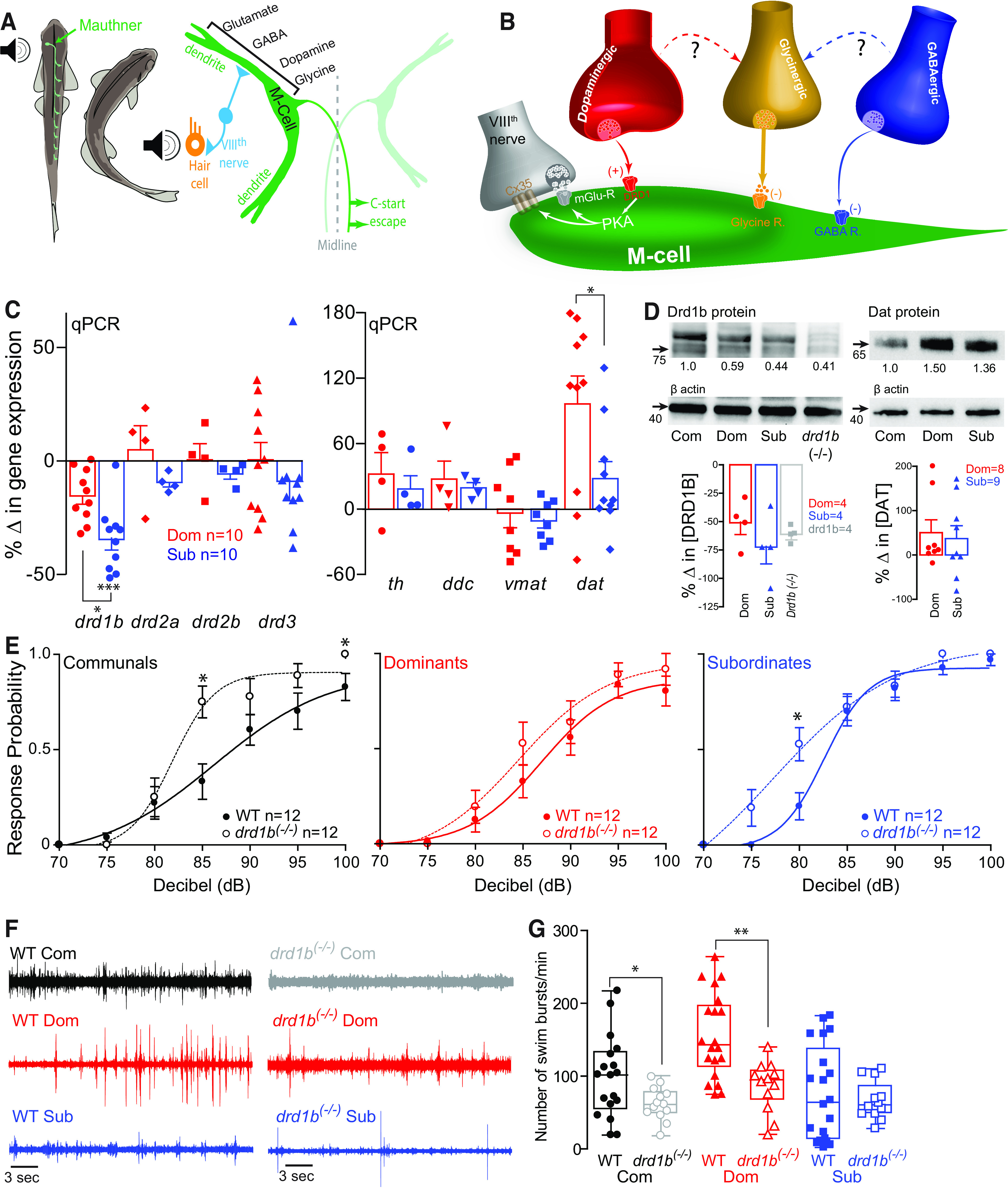
*drd1b* expression is socially regulated and necessary for status-dependent regulation of escape and swim circuits. ***A***, Schematic representation of the M-cell escape circuit and descending neuromodulatory inputs known to regulate its activity. ***B***, M-cell receives auditory sensory information from the VIIIth nerve, which is a mixed glutamatergic and electrical synapse. M-cell is also modulated by descending dopaminergic, glycinergic, and GABAergic inputs. ***C***, qPCR expression analysis of DA signaling pathway genes in whole brains (*n* = 10 pairs). β-Actin (*actb2*) used as an internal reference gene, and expression from social isolates (*n* = 10) used for standardization. Bars represent mean and SEM. Zero indicates no change in gene expression relative to isolate control. We performed one-way ANOVA (between-subject factor as group) followed by Tukey’s HSD *post hoc* test for the group comparisons to compare the percentage changes in gene expressions of *drd1b*, *drd2a*, *drd2b*, *drd3*, *th*, *ddc*, *vmat*, *dat* among isolates, dominants, and subordinates. In *drd1b*, there was a significant main effect of group (*F*_(2,32)_ = 1.93e+1, *p* = 3.00e-6). The *post hoc* test showed a significant decrease in gene expression for subordinates (*p* = 2.00e-6) and marginal decrease for dominants (*p* = 6.39e-2) compared with the isolates. The *post hoc* test also showed a significant decrease in gene expression for subordinates compared with dominants (*p* = 1.43e-3). In *drd2a*, *drd2b*, and *drd3*, there were no main effects of group (*p* > 0.05). In *th* and *vmat*, there were no effects of group (*p* > 0.05). In *ddc*, there was a main effect of group (*F*_(2,14)_ = 3.95, *p* = 4.35e-2). Dominant was higher than isolates (*p* = 4.61e-2). But there were no differences between dominants and subordinates (*p* > 0.05) and between subordinates and isolates (*p* > 0.05). In *dat*, there was a significant main effect of group (*F*_(2,30)_ = 8.71, *p* = 1.04e-3). The *post hoc* test showed a significant increase for dominants compared with isolates (*p* = 8.69e-4) and subordinates (*p* = 2.37e-2), but there was no difference between isolates and subordinates (*p* > 0.05). ***D***, Western blot analysis of Drd1b receptor and DAT with β-actin serving as a control. Protein expression of dominants, subordinates and *drd1b*^(−/−)^ animals was normalized to WT communal controls as a ratio (ratio values stated below each band). Bar graphs represent average percent change in protein concentration of four replicates of Drd1b samples and five replicates of DAT samples. Each replicate consisted of 10 brains normalized to WT communals. In Drd1b protein and Dat protein, there were no effects of group (*p* > 0.05; *n* = 4 replicates of dominants, subordinates, and *drd1b*^(−/−)^). ***E***, Comparison of the probability of the startle escape response between WT versus *drd1b*^(−/−)^ for each social phenotype. Asterisks denote statistical difference at the specified dB level. We performed a mixed design ANOVA (between-subject factor as group, within-subject factor as decibel) followed by two-sample two-sided *t* test for the *post hoc* test at each decibel. In communals, there were significant main effects of group (*F*_(1,22)_ = 4.73, *p* = 4.08e-2) and decibel (*F*_(3.5,76.3)_ = 5.85e+1, *p* < 1.0e-16), and group*decibel interaction (*F*_(3.5,76.3)_ = 3.21, *p* = 2.20e-2). We observed that *drd1b*^(−/−)^ significantly increased the startle response for communal animals. In particular, *post hoc* test showed that there were significant differences of the startle responses at 85 dB (*t*_(22)_ = 3.07, *p* = 5.57e-3), at 100 dB (*t*_(22)_ = 2.21, *p* = 3.75e-2), and marginal difference at 95 dB (*t*_(22)_ = 1.80, *p* = 8.58e-2). In dominants, there was a significant main effect of decibel (*F*_(3.5,76.4)_ = 5.54e+1, *p* < 1.0e-16), but no effect of group (*F*_(1,22)_ = 1.05e-1, *p* > 0.05) and no effect of group*decibel interaction (*F*_(3.5,76.4)_ = 2.83e-1, *p* > 0.05). In subordinates, there were significant main effects of group (*F*_(1,22)_ = 4.49, *p* = 4.57e-2), decibel (*F*_(3.6,78.4)_ = 1.00e+2, *p* < 1.0e-16), and marginal group*decibel interaction (*F*_(3.6,78.4)_ = 2.17, *p* = 8.79e-2). We observed that *drd1b*^(−/−)^ significantly increased the startle response for subordinate animals. In particular, *post hoc* test showed that there were significant differences of the startle responses at 80 dB (*t*_(22)_ = 2.63, *p* = 1.52e-2) and at 95 dB (*t*_(22)_ = 2.13, *p* = 4.48e-2), and a marginal difference of the startle response at 75 dB (*t*_(22)_ = 2.03, *p* = 5.48e-2). ***F***, Comparison of 1 min recordings of far field-potentials of spontaneous swimming activity for WT communals, dominants, and subordinates (left column) and *drd1b*^(−/−)^ communals, dominants, and subordinates (right column). ***G***, Box and whiskers plots of the average number of swim bursts per 1 min for each social phenotype for WT and *drd1b*^(−/−)^ animals. Dots represent individual animals. The box extends from the 25th to 75th percentiles, horizontal line is the median, and whiskers represent max/min values. We performed one-way ANOVA (between-subject factor as group) to compare WT and *drd1b*^(−/−)^ animals. In communals, there was a significant difference between WT (*n* = 18) and *drd1b*^(−/−)^ (*n* = 13) animals (*F*_(1,29)_ = 4.89, *p* = 3.50e-2). In dominants, there was a significant difference between WT (*n* = 20) and *drd1b*^(−/−)^ (*n* = 12) animals (*F*_(1,30)_ = 1.38e+1, *p* = 8.29e-4). In subordinates, there was no difference between WT (*n* = 20) and *drd1b*^(−/−)^ (*n* = 12) animals (*F*_(1,30)_ = 2.44e-1, *p* > 0.05; *p* values: *p < 0.05, **p < 0.005, ***p < 0.0005).

Zebrafish are social animals and agonistic interactions among conspecifics often culminate with the formation of stable dominance relationships. These social relationships are based on aggression, with highly ranked aggressive animals dominating low ranked subordinates (Oliveira et al., 2011; Teles and Oliveira, 2016; Clements et al., 2018). Prior work has shown that patterns of motor activities of the startle escape and swim circuits are socially determined (Miller et al., 2017; Park et al., 2018; Orr et al., 2021). As social dominance matures, dominant animals increase their swimming activities while subordinates reduce their swimming, but sensitivity of their startle escape response is significantly enhanced. However, the neuromodulatory mechanisms underlying this status-dependent switch in motor behavior remained unanswered. In teleost fish, the startle escape response is controlled by the Mauthner cells (M-cells), a pair of command neurons located in the hindbrain (Fig. 1*A*). Each M-cell receives ipsilateral auditory input from the VIIIth nerve and innervates contralateral fast motor neurons in the spinal cord (Eaton et al., 2001; Korn and Faber, 2005). Activation of the M-cell leads to the simultaneous activation of spinal fast motor neurons that control the startle reflex and inactivation of slow motor neurons that control swimming. The net effect is an uninterrupted startle escape response away from the perceived threat (Eaton et al., 2001). Conversely, swimming is coordinated by a distributed network of neurons in the brainstem and spinal cord. Swimming is initiated in the mesencephalic locomotor region, which innervates reticulospinal neurons in the hindbrain to activate central pattern generators in the spinal cord leading to a rhythmic oscillatory behavior (Roberts et al., 2008).

Both motor circuits receive local and descending excitatory and inhibitory modulatory inputs that control their excitability and activity phase transitions (Mu et al., 2012; Medan and Preuss, 2014; McPherson et al., 2016; Fig. 1*B*). One of these neuromodulators that regulates motivated behavior is the dopamine (DA; Wenzel and Cheer, 2018). In addition to excitatory inputs, descending GABAergic and glycinergic inputs provide tonic feedforward and backward inhibitory regulation of the M-cell for added control of motor activity (Oda et al., 1995; Hatta et al., 2001; Tabor et al., 2018). While the involvement of these three neuromodulators in regulating motor circuits has been investigated, the mechanism of how their synergistic modulation is affected by social factors to optimize motor output remains unknown. Here, we describe a mechanism by which social status induces cellular and biochemical plasticity in these neuromodulatory signaling pathways to regulate network excitability of the escape and swim circuits. Our empirical and neurocomputational results provide a novel account of how social experience induces functional plasticity on the cooperative action of multiple neuromodulators to control motor activity.

## Materials and Methods

### Animal maintenance

Adult zebrafish (*Danio rerio*) AB strain of mixed sex were housed communally in 10-liter tanks (∼20 animals) at the core animal facility and kept at a constant temperature at 28°C under a 14/10 h light/dark cycle. Fish were fed twice daily with high protein commercial food (Otohime B2, Reed Mariculture), and once daily with newly hatched artemia (Brine Shrimp Direct). All experiments were performed in accordance with the Institutional Animal Care and Use Committee.

### Social isolation and pairing

Only male zebrafish (6–12 months old) were selected from the group-housed (Communal tanks) and initially isolated physically and visually in individual tanks for one week. Following isolation, fish of equal size and age were paired in new tanks over a two-week period to establish a social dominance relationship. Aggressive interactions (number of attacks and retreats) were observed daily to determine and follow stability of social dominance as described elsewhere (Miller et al., 2017). Unless otherwise noted, only communal male zebrafish were selected from the group-housing tanks to serve as controls for the startle/swim, pharmacological and histologic analysis. Communal males were siblings (same genetic background) and same age as the experimental groups (dominants and submissive fish).

### Experimental setup

After the pairing phase was complete, fish were temporarily separated, and behavioral testing was performed sequentially. Each fish was placed in a testing chamber (dimensions: 11 × 4 × 3 cm) filled with double distilled water having a resistance of ∼15 MΩ-cm. High-resistance water increases signal-to-noise ratio and was shown to have no adverse effects on the health of the fish (Issa et al., 2011). A pair of conductive electrodes placed on either side of the chamber recorded the electric field potentials. Bare electrodes were 1 mm in thickness with 3- to 5-mm metal exposure. Electrodes were connected to an AC differential amplifier (A-M Systems model 1700), allowing the amplification of signals 1000-fold. Electrical signals were low-pass filtered at 300 Hz and high-pass filtered at 1 kHz. Signals were digitized using a Digidata-1322A digitizer and stored using Axoscope software (Molecular Devices). The animals were acclimatized for 30 min before behavioral testing was initiated. Swimming behavior was recorded following acclimation. Immediately after, startle responses were taken and recorded.

### Determination of startle sensitivity

Auditory pulses consisting of phasic 1-ms square waves were generated using Audacity open-source audio editor and recorder software (https://www.audacityteam.org/). Sound pulses were amplified with (Realistic, SA-10 solid state stereo amplifier) and connected to a sound speaker (Dell, HK395). Sound intensity was calibrated using a decibel meter (Sinometer, MS6700). Activation of the Mauthner-mediated escape is an all or nothing response with a short latency from stimulus (5–15 ms), and startles were only included if they fell within this range. Non-Mauthner mediated responses >15 ms were excluded (Eaton et al., 2001). Pulse intensity ranged from 70–100 dB re 20 μPa with 5-dB increments and delivery was randomized with a minimum of 2-min intervals between stimuli. Each pulse intensity was delivered three to five times, and response probability for each intensity was tabulated then averaged across animals.

### Measurement of swimming activity

Following the 30-min acclimation period and before determination of startle sensitivity, the animal’s swimming behavior was recorded for 1 min. The same methods of data acquisition, amplification, digitization, and storage were used as previously stated. Swimming activity was measured by counting swim bursts with Clampfit software. The “Threshold” function was used for this purpose. A potential was marked as a swim burst if it was at least 8 mV in total amplitude and 30–200 ms in duration. The timing of each swim burst was saved into a Microsoft Excel spreadsheet in reference to the recording start time.

### Data analysis and statistics

Startle and swimming data were analyzed using Prism software (GraphPad Inc.) and IBM-SPSS (RRID:SCR_002865). Unless specified otherwise, all comparisons were first subjected to one-way ANOVA (between-subjects factor as group) or mixed design (a mixture of between-subjects factor and repeated measures variables) ANOVA (between-subjects factor as group; within-subjects factor as decibel) or repeated measures ANOVA (within-subjects factors as treatment and decibel) followed by Tukey’s HSD *post hoc* test or two-sided *t* test *post hoc* test for all multiple comparisons. Before using the mixed design or repeated measures ANOVA, sphericity was tested by using Mauchly’s test. When the assumption of sphericity was violated, the degree of freedom in Greenhouse–Geisser correction was used. The one-way ANOVA is considered a relatively robust test against the normality assumption. Startle data were initially curve fitted to multiple equations to determine best fit and highest *R*^2^ value. The Boltzmann equation produced the best fit with the highest *R*^2^ values. Thus, nonlinear regressions were performed and fitted using the Boltzmann sigmoidal equation: Y=Bottom+(Top−Bottom)/(1+exp((V50−X)/Slope)).

### Pharmacology

Control experiments were performed on day 14 postpairing during which startle and swimming behaviors were tested and measured. Next day (day 15) fish were injected intramuscularly with the appropriate drug and their startle and swimming behaviors measured again. Immediately after injections the pairs were returned to their pairing tanks but separated with a divider for a period of 1.5 h post-injection of recovery. After the recovery period, the animals were transferred to the behavioral testing chamber and given 30 min of acclimation period.

For injection, glass capillaries were used, having the dimensions 1.0 mm outer diameter (OD) × 0.5 mm inner diameter (ID) × 100 mm in length. These were pulled using Flaming/Brown Micropipette Puller (Model P-87, Sutter Instrument Co). The tip of the micropipette was broken off with a razor blade, before loading with the drug solution. Loaded micropipettes were placed in Pneumatic PicoPump PV 820 for drug administration. A 0.02% tricaine solution was used to anaesthetize the animal before injection. A stock solution of 20 mm each drug was prepared at a concentration of 5 mg/ml. Based on the drugs’ molecular weights, the final concentrations injected were: 25 mm for L-DOPA, 15 mm for SCH23390 (D1 antagonist), 10 mm for bicuculline methobromide (GABA antagonist), and 15 mm for strychnine [glycine receptor (GlyR) antagonist]. These concentrations fall within range of prior reports and our preliminary testing whereby administration of higher concentrations (10 mg/ml) led to increased behavioral deficits (D1 antagonist, bicuculine) and paralysis (strychnine), and prior reports showed that concentration of ≤1.5 mg/ml the drugs had no detrimental effects on motor activity (Roy et al., 2012). All drugs were purchased from Tocris Bioscience except Strychnine, which was purchased from VWR.

### Dopamine signaling molecules RNA extraction

RNA extractions were performed using a modified phenol-chloroform protocol using Trizol reagent. Zebrafish were anesthetized with 0.02% MS-222 then euthanized in iced water (10 min). This was followed by dissection and extraction of the brains which were placed in 2-ml microtubes and homogenized by gentle sonication on ice, 400 μl of additional TRIzol was added. The homogenized whole-brain samples were incubated at room temperature for 5 min. A total of 160 μl of chloroform was added, and the samples were mixed. Samples were incubated at 4°C for 20 min with intermittent mixing, followed by centrifugation. The aqueous phase was transferred to a new, sterile tube, and 500-μl ice-cold ethanol was added. The sample was loaded onto a RNeasy spin column (QIAGEN) and spun. Samples were washed with buffer RW1, followed by two washes of Buffer RPE. RNA was eluted with two, 20-μl volumes of RNase-free water into an RNase-free Microfuge tube. RNA extracts were quantified by Nanodrop 2000 (Thermo Scientific), and then stored at −80°C until ready to use.

### RT synthesis of cDNA stocks

QIAGEN’s QuantiText Reverse Transcription kit was used for cDNA synthesis of RNA extracts. RNA was brought to a volume of 12 μl with RNase-free water, and 2 μl QIAGEN gDNA wipeout buffer was added. Samples were incubated at 42°C for 2 min to eliminate any DNA contamination. Samples were incubated at 42°C for 30 min for cDNA synthesis, then 3 min at 95°C for enzyme deactivation. Samples were brought up to a final RNA concentration of 10 ng/μl with RNase-free water and stored at −20°C until ready for qPCR.

### qPCR primer design and validation

Primers were designed using NCBI Primer-BLAST software and are summarized in Table 1. All primers were designed to span an exon-exon junction, minimize secondary products, produce an amplicon of 75–150 bp, and have an annealing temperature of 60–67°C. Primers were validated to confirm amplicon size, and to determine ideal primer concentration for qPCR reactions. 20 μl PCR reactions were run using GoTaq Flexi DNA Polymerase Reagents from Promega. Primer pairs were assessed at concentrations of 500, 750, and 900 nm final concentrations for amplicon amounts, and the presence of primer-dimer secondary product. Samples were run on a 2% agarose gels containing 0.5× GelRed for visualization under UV light, and pictures were taken with Alpha Innotech’s FluorChem 8900 gel imaging system. Working primer concentrations were determined to be: 500 nm for all primer pairs.

**Table 1:** Primers for qPCR analysis

Gene	Sequence	Tm (°C)	Amplicon size (bp)	Primer efficiency
*actb2*	Fw: CCAAACCCAAGTTCAGCCATGG	62.25	118	99.1%
	Rv: TGGATGGGAAGACAGCACGG	62.18		
*th*	Fw: TTCAGCCATACCAAGACCAG	61.6	147	109.4%
	Rv: CTTCTATGCTGTCCGTGTACG	61.2		
*ddc*	Fw: ACCCTGATGTGGAACCGGGA	63.05	137	131.7%
	Rv: GTATGGGCTGTGCCAGTGGG	63.18		
*slc18a2* (vmat)	Fw: CGGAGGCTGATTCTGTTGATCG	61.37	120	106.4%
	Rv: CATCTGAGCAGCCTCGTCGT	62.28		
*slc6a3* (dat)	Fw: AAACACAACGTGGCCCTGGA	62.56	108	90.9%
	Rv: GACTGCCCACACCGAAGAGC	63.39		
*drd1b*	Fw: TGACAAGGTCTGTGGGAGTACA	63.4	147	104.2%
	Rv: ACGTGAATCGGAGCAACTGG	66.3		
*drd2a*	Fw: CCTCCATTGTGTCCTTCTACG	62.3	123	93.7%
	Rv: TGTCTGTAACTGGGCATGTG	61.2		
*drd2b*	Fw: GCTTTCATTCGCCATTTCCTG	66.5	142	93.6%
	Rv: GGTGATAATGAAGGGCACGTAG	63.3		
*drd3*	Fw: TCTTTGTGACCCTGGATGTG	62.5	140	92.1%
	Rv: CATGACTGAAACCCTTTTGCG	65.4		

List of genes and associated designed primers for qPCR analysis. Gene names are italicized: actin, β2 (*actb2*); tyrosine hydroxylase (*th*); DOPA decarboxylase (*ddc*), Synaptic vesicle monoamine transporter (*vmat*), dopamine transporter (*dat*), dopamine receptor type Ib (*drd1b*), dopamine receptor type 2a (*drd2a*), dopamine receptor type 3 (*drd3*).

### qPCR primer efficiency determination

Standard curves for primer efficiencies were prepared using an initial cDNA amount of 30-ng total cDNA, then 1:10 dilutions were prepared. 20 μl reactions of each cDNA amount and each primer pair were run in triplicate on a 96-well PCR plate from Applied Biosystems. Primer pairs were used in predetermined optimal concentrations, and 10 μl 2× Applied Biosystems Fast SYBR green reaction mix was added for a total volume of 20 μl. Real-time PCR reactions were run on an Applied Biosystems Quantistudio 12k Flex Real-Time PCR System for Ct determination. Primer efficiency standard curves were prepared using the Quantistudio 12k Flex Real-Time PCR System software and are reported in Table 1.

### qPCR reaction protocol

qPCR reactions were run using Applied Biosystems Fast SYBR Green Master mix, using Applied Biosystems Quantiostudio 12k Flex Real-Time PCR System. 96 well plates were used with duplicate technical replicates, and 20 μl reactions were prepared. SYBR green detection occurred during each cycle’s amplification stage. Passive reference dye ROX was used for volume standardization. All samples were compared with internal housekeeping gene *actb2* for normalization, and isolate fish served as a biological control. Three biological replicates of each social group (dominant, subordinate, and isolate) were analyzed on each 96-well plate. Multiple 96-well plates were run so that each gene had *n* = 10.

### qPCR data analysis

qPCR data were analyzed using the comparative ΔΔCt method. Ct values were determined for each gene of each biological replicate, and experimental genes were compared with internal housekeeping gene *actb2* for determination of ΔCt values. ΔΔCt values were determined by normalization of dominant and subordinate social groups to social isolate controls, and percent change was determined from ΔΔCt values using the appropriate primer efficiencies. Percent-change values were transformed to a log base 2 scale for graphing and statistical analysis. Genes percent changes were analyzed for significance by two-sided one sample *t* test from a theoretical mean of 0, while percent changes between social groups (dominant and subordinate) were analyzed using a two-sided Wilcoxon signed rank test, α = 0.05.

### Western blotting

For Western blot analysis four separate Western blot trials were conducted with 10 brains per trial for each social phenotype. Zebrafish were anesthetized with 0.02% MS-222 (1 min) then placed in iced water (10 min). Brains were dissected out, placed in a 1.5-ml microcentrifuge tube, and stored at −20°C until use. Then they were prepared using the total membrane isolation protocol. Brains were homogenized in 1 ml of resuspension buffer: 2.5 ml 2 m sucrose, 2 ml 10 × 10 mm Tris-HCl, 400 μl 0.25 m EDTA, 40 μl 20× protease cocktail inhibitor. The homogenized brains were centrifuged (2000 rpm, 4°C, 10 min) followed by ultracentrifugation (37,000 rpm, 4°C, 60 min). Protein sample concentrations were determined with a Lowery protein assay.

For Western blottings, 10 μg of each protein sample was denatured using 4× buffer containing 10× reducing reagent at 70°C for 10 min and loaded onto a Mini-PROTEAN TGX Precast Protein Gels (Bio-Rad) and run ∼120min at 60V. The proteins were transferred to a 0.2 μm Nitrocellulose membrane using Trans-Blot Turbo RTA Mni Transfer kit (Bio-Rad). The membrane was blocked with PBS containing 5% (w/v) nonfat dry milk and 0.1% (v/v) Tween-20 for 1 h at room temperature and then incubated with primary antibodies overnight at 4°C. After three washes with PBS containing 0.1% (v/v) Tween-20, the membrane was incubated with horseradish peroxidase (HRP)-conjugated goat anti-mouse or goat anti-rabbit IgG (Cell Signaling) secondary antibody for 1 h. After washing, protein detection was performed using HRP detection kit (SuperSignal West Pico, Thermo Fisher Scientific) and visualized using the ChemiDoc Imaging System (Bio-Rad). Bands were quantified with ImageLab software and normalized to β-actin (Bio-Rad). Primary antibodies used were as follows: mouse anti-Drd1b (1:500; Novus Biological), Rabbit anti-DAT (1:500; ProteinTech Group Inc.), β-actin (1:1000; Cell Signaling).

### Histology, imaging, and analysis

To determine differences in Drd1b expression in hindbrain glycinergic neurons between social phenotypes, we used the [Tg*(GlyT2a:GFP*)] transgenic line kindly provided by the Fetcho lab (McLean et al., 2007). Animals’ sex, size, social isolation, pairing, and behavioral analysis were conducted as described above. Animals were then euthanized in a heavy dose of MS-222 (0.25% by volume). Animals were pinned on Sylgard lined Petri dish (Dow Corning) and brains extracted. Brains were fixed overnight in 4% PFA at 4°C, washed 3 × 5 min in 0.1% PBS-T then placed in 30% sucrose for an additional night at 4°C. Brains were then placed in plastic molds containing O.C.T. tissue compound (Tissue-Tek) and frozen in liquid nitrogen and stored at −20°C. Frozen brains were sliced into 60-μm slices using a cryostat (Leica Biosystems) and mounted on Superfrost plus slides (Fisher Brand). Brain slices were incubated in a blocking buffer for 2 h, followed by an incubation in a Rabbit polyclonal Drd1b primary antibody (ThermoFisher, catalog #PA5-33477) 1:500 overnight at 4°C. Primary antibody was washed in PBS 3 × 5 min, then incubated in an Alexa-555 goat anti-rabbit secondary antibody (Invitrogen, catalog #A21428) 1:1000, finally washed in PBS 3 × 5 min. Slices then were mounted with Antifade reagent and cover slip, then sealed 24 h later. All images were acquired with a 40× oil immersion using Carl Zeiss LSM 800 upright confocal microscope whose acquisition parameters were kept similar across experimental conditions.

### Confocal image analysis

All acquired images from experimental and control groups were coded and analyzed blindly. Hindbrain glycinergic neurons were identified visually based on their morphologic location in relation to spinal midline and M-cell location. All glycinergic and drd1b expressing neurons were quantified using Imaris software (version 9.3; Oxford Instruments plc) surface and spots functions. This was followed by identification and quantification of all glycinergic neurons that specifically express the Drd1b using the Imaris co-localization function. Ratios of Drd1b expressing cells to total number of glycinergic neurons was tallied and data plotted using GraphPad Prism software. Representative confocal images were imported into Adobe Photoshop, stack projections (60-μm slices) were generated, and dust and scratches noise filter was applied to red channel of all images to eliminate background noise.

*drd1b*^(−/−)^ knock-out (KO) line was kindly provided by Teresa Nicolson (Stanford University) and originally created at the Wellcome Sanger Institute (Cambridgeshire, United Kingdom). The mutant line (allele: sa16476) contains a nonsense point mutation (G > A) in the second exon on chromosome 9 that affects amino acid number 314 leading to a truncated nonfunctional protein (Ensembl ID: ENSDARG00000038918); ZFIN ID: ZDB-GENE-070524-2.

### Neurocomputational model

In the present study, we consider the behavior of M-cell that receives excitatory and inhibitory inputs, which is modulated by dopamine. The model network is composed of one excitatory neuron, two inhibitory neurons (GABAergic and glycinergic), and one M-cell. The excitatory neuron receives sensory inputs and excites all the other neurons. GABAergic neuron inhibits glycinergic neuron and M-cell. Glycinergic neuron inhibits M-cell only.

All neurons were modeled as a conductance-based modified Morris–Lecar neuronal model (Morris and Lecar, 1981; Izhikevich, 2007; G.B. Ermentrout and Terman, 2010) with additional calcium-dependent potassium current. The membrane potential of each cell obeys the following current balance equation:

$$C\frac{dv}{dt}=-I_{Ca}-I_{K}-I_{L}-I_{KCa}-I_{syn}+I_{app}(t),$$

where $I_{K}=g_{K}n\left(v-v_{K}\right),$ $I_{Ca}=g_{Ca}m_{\infty }\left(v\right)\left(v-v_{Ca}\right),$ $I_{KCa}=g_{KCa}\left\{\frac{\left[Ca\right]}{\left[Ca\right]+k_{1}}\right\}\left(v-v_{K}\right),I_{L}=g_{L}\left(v-v_{L}\right)$ represent the potassium, calcium, calcium-dependent potassium, and leak currents, respectively. $I_{syn}$ represents the synaptic currents and $I_{app}(t)$ is the applied current (see below). [Ca] represents intracellular calcium concentration. For all neurons, $g_{K}=8$, $v_{K}=-84$, $g_{L}=2$, $v_{L}=-60$, $g_{Ca}=4$, $v_{Ca}=120$, and $k_{1}=10$. In M-cell, $g_{KCa}=0.3$ and C=1. In other neurons, $g_{KCa}=0.25$ and C=20.

$m_{\infty }$ is an instantaneous voltage-dependent gating variable for the calcium current where

$$m_{\infty }\left(v\right)=0.5\left(1+\tanh\left(\frac{v-v_{1}}{v_{2}}\right)\right),$$

with $v_{1}=-1.2$ and $v_{2}=18.$

The concentration of intracellular $Ca^{2+}$ is governed by the calcium balance equation

$$\frac{d\left[Ca\right]}{dt}=\text{ε}\left(-\text{μ }I_{Ca}-k_{Ca}\left[Ca\right]\right),$$

where ε=0.005,  μ=0.19. In M-cell, $k_{Ca}=0.9$ while $k_{Ca}=1$ in all other neurons.

n is a gating variable for the potassium current obeying

$$\frac{dn}{dt}=\frac{\phi \left(n_{\infty }\left(v\right)-n\right)}{\tau_{n}\left(v\right)},$$

$$n_{\infty }\left(v\right)=0.5\left(1+\tanh\left(\frac{v-v_{3}}{v_{4}}\right)\right),$$

$$\tau_{n}\left(v\right)=1/\text{cosh}\left(\frac{v-v_{3}}{2v_{4}}\right),$$

where ϕ=0.23, $v_{3}=12$, and $v_{4}=17$.

In an excitatory cell and inhibitory cells, synaptic variable, *s*, is modeled by an equation for the fraction of activated channel

$$\frac{ds}{dt}=\alpha s_{\infty }\left(v\right)\left(1-s\right)-\beta s,$$

where $s_{\infty }\left(v\right)=1/\left(1+\exp\left(-\frac{v+\theta_{s}}{\sigma_{s}}\right)\right)$ with $\theta_{s}=0$ and $\sigma_{s}=4$. Here, α=15 and β=0.1 in an excitatory cell, α=4 and β=0.08 in a GABAergic cell, and α=8 and β=0.08 in a glycinergic cell.

The synaptic current ( $I_{syn}$), the sum of synaptic inputs from other cells, is given by

$$I_{syn}=g_{syn}(v-v_{syn})\underset{j}{\sum }s_{j},$$

where the summation is over s variables from all neurons projecting to a given neuron.

In the current neuronal network, an excitatory cell does not receive any synaptic input but receives an external stimulus to simulate the effect from the hair cell or VIIIth nerve fiber. Thus, in an excitatory cell, $I_{syn}=0$ and $I_{app}\left(t\right)=I_{E0}+W_{E}*I\left(\tau \right)$, where $I_{E0}$ is a baseline external input, $W_{E}$ is the stimulus strength and I(τ) is the stimulus at time τ. $I_{E0}=43.9$ and $W_{E}\in \{10,15,20,...,55,60\}$ in the model. Here, I(τ) resembles the unit square pulse with height 1.

GABAergic cell does not receive any external stimulus but receives a synaptic input from the excitatory cell. Thus, in a GABAergic cell, $I_{syn}=g_{\text{E}\to \text{GA}}\left(v-v_{\text{E}\to \text{GA}}\right)s_{E}$, where $s_{E}$ is the synaptic variable from the excitatory cell and $I_{app}\left(t\right)=I_{GA0}=36$, a baseline external input to the GABAergic cell. We use $v_{\text{E}\to \text{GA}}=40\text{ and }g_{\text{E}\to \text{GA}}=0.3$ in the model.

Glycinergic cell receives synaptic inputs from the excitatory cell and the GABAergic cell. Thus, in glycinergic cell, $I_{syn}=g_{\text{E}\to \text{GL}}\left(v-v_{\text{E}\to \text{GL}}\right)s_{E}+g_{\text{GA}\to \text{GL}}\left(v-v_{\text{GA}\to \text{GL}}\right)s_{GA}$, where $s_{E}$ is a synaptic variable from the excitatory cell and $s_{GA}$ is a synaptic variable from GABAergic cell. $I_{app}\left(t\right)=I_{GL0}=36$, a baseline external input to the glycinergic cell. To reflect dopaminergic modulation over excitatory connection in the model, the excitatory maximal synaptic conductance ( $g_{\text{E}\to \text{GL}}$) was modified as explained below.

In M-cell, which receives synaptic inputs from all the other cells in the network,

$$I_{syn}=g_{\text{E}\to \text{M}}\left(v-v_{\text{E}\to \text{M}}\right)s_{E}+(g_{\text{GA}\to \text{M}}s_{GA}+g_{\text{GL}\to \text{M}}s_{GL})\left(v-v_{\text{G}\to \text{M}}\right)+g_{\text{M}\to \text{M}}\left(v-v_{\text{M}\to \text{M}}\right)s_{M},$$

where $s_{M}$ is the synaptic variable from another M-cell, which is assumed to be a small constant ( $s_{M}=0.029)$ in the current study. $s_{GL}$ is a synaptic variable from the glycinergic cell. $I_{app}\left(t\right)=I_{M0}=19.5$, a baseline external input to the M-cell. Now, calcium is known to modulate inhibitory presynaptic neurotransmitter release via retrograde signaling (Diana and Bregestovski, 2005). In the model M-cell, we assumed that intracellular calcium level reciprocally modulates the presynaptic inputs to the M-cell and $I_{syn}$ is updated as follows:

$$I_{syn}=g_{\text{E}\to \text{M}}g_{\text{I}}\left(v-v_{\text{E}\to \text{M}}\right)s_{E}+g_{\text{I}}(g_{\text{GA}\to \text{M}}s_{GA}+g_{\text{GL}\to \text{M}}s_{GL})\left(v-v_{\text{G}\to \text{M}}\right)+g_{\text{M}\to \text{M}}g_{\text{I}}\left(v-v_{\text{M}\to \text{M}}\right)s_{M},$$

where $g_{\text{I}}$ obeys the following equation

$$\frac{dg_{I}}{dt}=\frac{\frac{g_{Imax}}{[Ca]+k_{2}}-g_{I}}{\rho },$$

where $g_{Imax}$ is the maximal $g_{I}$ value, ρ is the time constant of $g_{I}$, and [Ca] is the intracellular calcium concentration of the M-cell. Here, parameter values are $g_{\text{E}\to \text{M}}=0.24,\;v_{\text{E}\to \text{M}}=40,\;v_{\text{G}\to \text{M}}=-50,\;g_{\text{M}\to \text{M}}=0.1,\;v_{\text{M}\to \text{M}}=-50,\;g_{Imax}=15,k_{2}=10,\rho =10000,g_{\text{GA}\to \text{M}}=0.4,g_{\text{GL}\to \text{M}}=0.2$. Now we implemented the dopamine modulation of synaptic inputs in the glycinergic cell, and the M-cell. In the current network, we assume that D_1_ enhances the input that a cell receives.

In the glycinergic cell, the excitatory input is enhanced by D_1_, which is reflected in $I_{syn}$ as follows:

$$I_{syn}=g_{\text{E}\to \text{GL}}(1+D_{1GL})\left(v-v_{\text{E}\to \text{GL}}\right)s_{E}+g_{\text{GA}\to \text{GL}}\left(v-v_{\text{GA}\to \text{GL}}\right)s_{GA},$$

where $g_{\text{E}\to \text{GL}}=0.3,v_{\text{E}\to \text{GL}}=40$, and $v_{\text{GA}\to \text{GL}}=-50.$ We also used $D_{1GL}=0.65$ and $g_{\text{GA}\to \text{GL}}=0.2$ for dominant-like model, and $D_{1GL}=0.25$ and $g_{\text{GA}\to \text{GL}}=0.4$ for subordinate-like model.

Lastly, in the M-cell, D_1_ enhances the amplitudes of excitatory input, which is reflected in $I_{syn}$ as follows:

$$I_{syn}=g_{\text{E}\to \text{M}}g_{\text{I}}\left(1+D_{1M}\right)\left(v-v_{\text{E}\to \text{M}}\right)s_{E}+g_{\text{I}}(g_{\text{GA}\to \text{M}}s_{GA}+g_{\text{GL}\to \text{M}}s_{GL})\left(v-v_{\text{G}\to \text{M}}\right)+g_{\text{M}\to \text{M}}g_{\text{I}}\left(v-v_{\text{M}\to \text{M}}\right)s_{M}$$

where $D_{1M}=0.015$.

In summary, we varied three parameters ( $D_{1GL}$, $g_{\text{GA}\to \text{GL}}$, β) between dominant-like model and subordinate-like model: $D_{1GL}=0.65$, $g_{\text{GA}\to \text{GL}}=0.2$ for dominant-like model and $D_{1GL}=0.25,$ $g_{\text{GA}\to \text{GL}}=0.4$ for subordinate-like model. We also varied the decay time constant β in the synaptic variable for the glycinergic input to simulate the blockage of the GABAergic receptor: β=0.024 for dominant-like model and β=0.0072 for subordinate-like model. These parameters were chosen based on the model hypothesis that dominant-like model has stronger dopaminergic→glycinergic→M, while subordinate-like model has stronger GABAergic→glycinergic pathways.

Simulations were performed on a personal computer using the software XPP (B. Ermentrout, 2002). The numerical method used was an adaptive-step fourth order Runge–Kutta method with a step size 0.01 ms.

### Code accessibility

The code described is deposited and freely available online at https://modeldb.science/2015414 and the code is available as Extended Data 1.

10.1523/ENEURO.0311-23.2023.supplement Extended Data 1 Code of the neurocomputational model including all the computational parameters utilized to develop the computational described. Download Supplementary 1, TXT file

## Results

To determine the effects of social status on DA signaling and spinal motor activity, we measured changes in expression patterns of genes involved in DA signaling. We found that expression of *drd1b* was significantly reduced in subordinates relative to dominants and control social isolates (Fig. 1*C*). Conversely, levels of dopamine reuptake transporter (*dat*) were significantly elevated in dominants compared with subordinates and control isolates; while DOPA Decarboxylase (*ddc)* expression was significantly higher in dominants compared only to control isolates. Western blot analysis of protein expression mirrored the mRNA results for the Drd1b, while differences in Dat protein expression between dominants and subordinates were present but less obvious (Fig. 1*D*). The observed differences in *drd1b* gene expression led us to examine whether the differences in the activation of DA signaling pathways underlie status-dependent motor activity. We postulated that if the increase in startle sensitivity in subordinates is because of the decrease in Drd1b expression then its blockage should elevate startle sensitivity. Indeed, we found that the startle sensitivity in *drd1b*^(−/−)^ communal and subordinate animals was significantly higher compared with wild-type (WT) animals, but not so for dominant animals (Fig. 1*E*). Moreover, swimming activity of the *drd1b*^(−/−)^ was significantly lower than that of WT communal and dominant animals (Fig. 1*F*,*G*).

### Dopaminergic modulation of motor circuits is socially regulated

**Figure 2.**
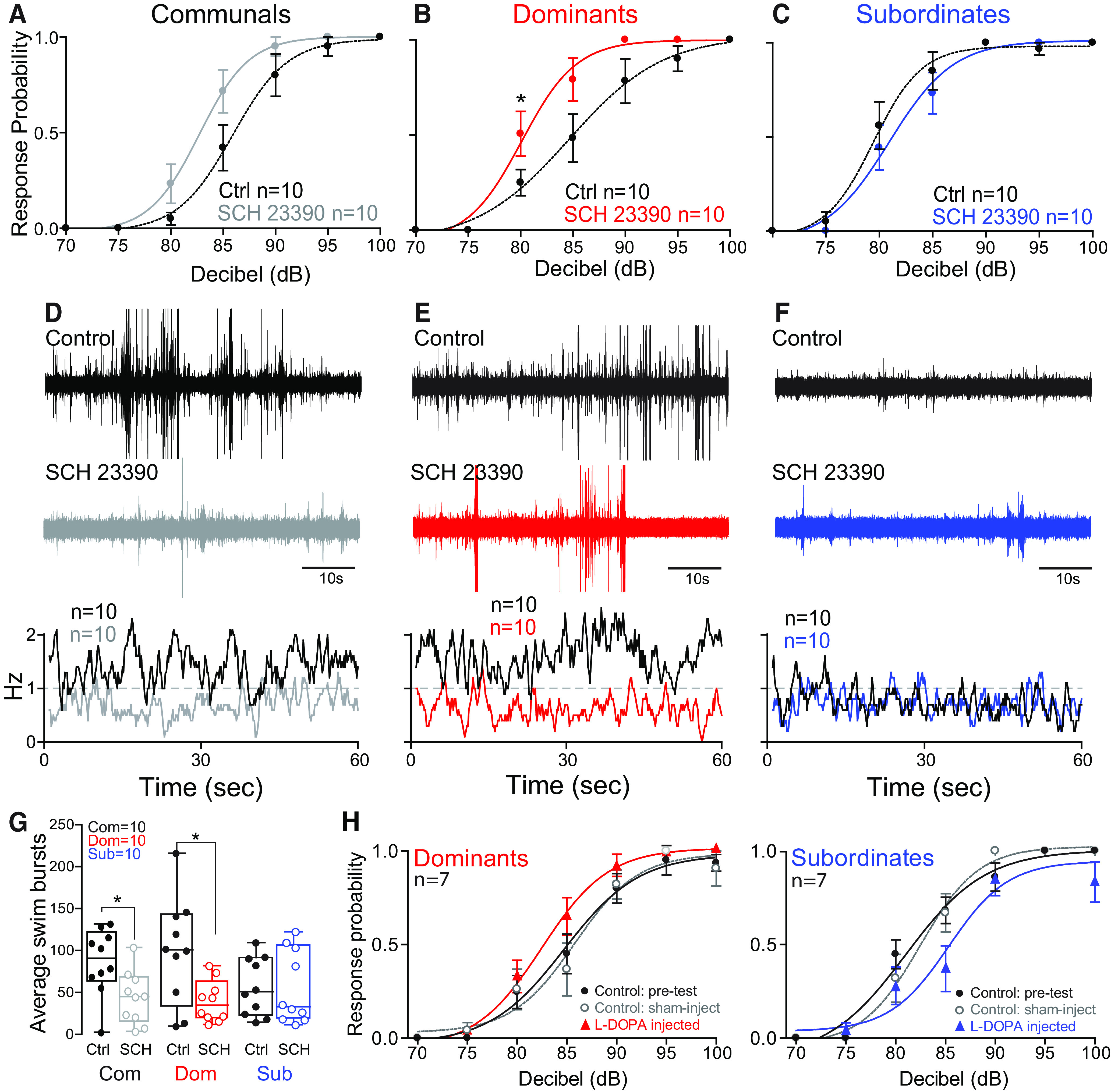
Dopaminergic modulation of the escape and swim circuits is socially regulated. ***A–C***, Probability of startle escape response before (control) and after SCH 23390 injections for communals, dominants, and subordinates, respectively. Asterisks denote statistical difference between control and experimental condition at the specified dB level (*p < 0.05, paired sample *t* test). We performed repeated measures of ANOVA (within-subject factors as treatment and decibel) followed by one-sample two-sided *t* test for the *post hoc* test at each decibel. In communals, there were significant main effects of treatment (*F*_(1,9)_ = 9.55, *p* = 1.29e-2) and decibel (*F*_(2.0,17.9)_ = 9.87e+1, *p* = 2.21e-10), but no effect of treatment*decibel interaction (*F*_(2.3,20.8)_ = 1.69, *p* > 0.05). SCH 23390 significantly increased the overall startle response for communal animals. In particular, *post hoc* test showed that there was a marginal difference of the startle responses at 90 dB (*t*_(9)_ = 1.96, *p* = 8.11e-2). In dominants, there were significant main effects of decibel (*F*_(1.5,13.9)_ = 7.08e+1, *p* = 1.39e-7), treatment (*F*_(1,9)_ = 1.44e+1, *p* = 4.24e-3), and a marginal treatment*decibel interaction (*F*_(1.8,16.0)_ = 2.81, *p* = 9.50e-2). SCH 23390 significantly increased the startle response for dominant animals. In particular, *post hoc* test showed that there was a significant difference of the startle response at 80 dB (*t*_(9)_ = 3.02, *p* = 1.44e-2), and marginal differences at 85 dB (*t*_(9)_ = 2.20, *p* = 5.51e-2) and at 90 dB (*t*_(9)_ = 1.86, *p* = 9.63e-2). In subordinates, there was a significant main effect of decibel (*F*_(1.4,12.4)_ = 9.72e+1, *p* = 9.37e-8), but no effect of treatment (*F*_(1,9)_ = 8.77e-1, *p* > 0.05) and no effect of treatment*decibel interaction (*F*_(1.9,17.0)_ = 8.81e-1, *p* > 0.05). ***D–F***, One-minute recoding of far field-potentials of spontaneous swimming activity before (control) and after SCH 23390 injections for communal, dominants, and subordinates, respectively, along with respective average swimming frequency for all animals tested (horizontal dashed lines set arbitrarily to compare swim frequencies across experimental conditions). ***G***, Box and whiskers plots of the average number of swim bursts per 1 min for each social phenotype. Box plot parameters are defined in Figure 1*G*. We performed the repeated measures of ANOVA (within-subject factor as treatment). In communals and dominants, there was a significant effect of treatment (SCH 23390; *F*_(1,9)_ = 6.06, *p* = 3.61e-2 for communals; *F*_(1,9)_ = 1.06e+1, *p* = 9.88e-3 for dominants). In subordinates, there was no effect of treatment (SCH 23390; *F*_(1,9)_ = 8.76e-3, *p* > 0.05). ***H***, Startle response probability before (control) and after injection of L-DOPA for dominant (left) and subordinate (right) zebrafish. Control and experimental data are compared with a second set of control animals that were sham injected with equal volume of reverse osmosis water.

The display of subordinate-like motor activity in communal and dominant *drd1b*^(−/−)^ animals led us to closely examine *drd1b* function in mediating the observed shifts in motor activity. We found that pharmacological blockage of the Drd1b receptor using the selective antagonist (SCH 23390) moderately increased startle sensitivity in communals and significantly increased startle sensitivity in dominants with no observable change in subordinates (Fig. 2*A–C*). Conversely, SCH 23390 significantly reduced swimming in communals and dominants while subordinates showed no change (Fig. 2*D–G*). These results show that either pharmacological blocking or genetic deletion of *drd1b* function promotes subordinate-like motor behavior (Figs. 1*E,G*, 2).

Differences in *dat* and *ddc* gene expression levels suggested that DA availability might work in concert with Drd1b in regulating motor activity. We addressed this possibility by augmenting DA levels via injection of L-DOPA. We found that L-DOPA led to opposing effects on startle sensitivity in the two social phenotypes (Fig. 2*H*). In dominants, L-DOPA moderately increased startle sensitivity, while in subordinates startle sensitivity was moderately decreased. Collectively, these results indicate that presynaptic and postsynaptic regulation in DA signaling pathway regulate M-cell excitability in a socially dependent manner.

### GABAergic modulation of M-cell excitability is socially regulated

**Figure 3.**
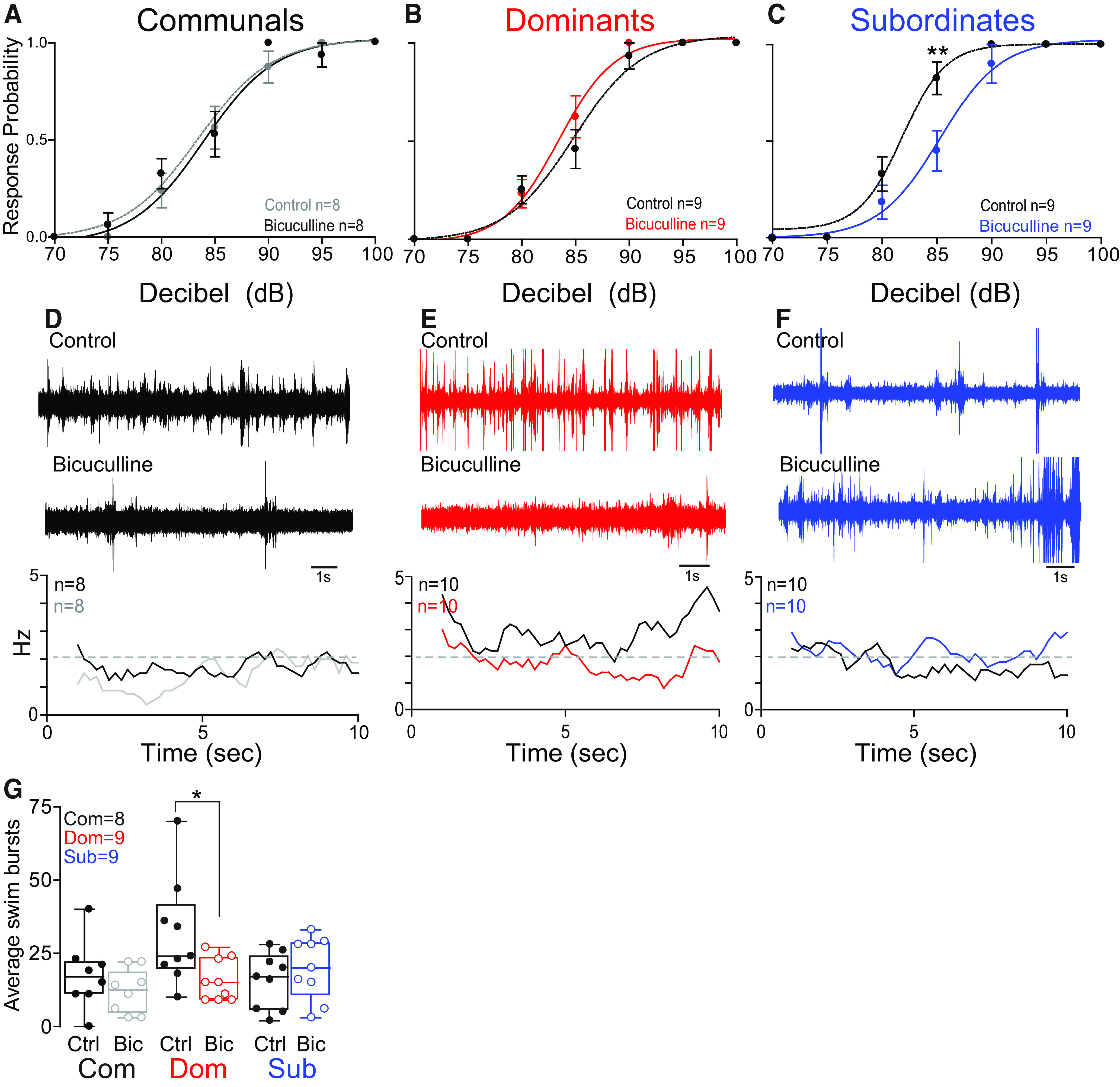
GABAergic modulation of the escape and swim circuits is socially regulated. ***A–C***, Probability of startle escape response before (control) and after bicuculline injections for communals, dominants, and subordinates, respectively. We performed repeated measures of ANOVA (within-subject factors as treatment and decibel) followed by one-sample two-sided *t* test for the *post hoc* test at each decibel. In communals, there was a significant main effect of decibel (*F*_(2.6,18.1)_ = 1.01e+2, *p* = 3.49e-11), but no effect of treatment (*F*_(1,7)_ = 2.80e-1, *p* > 0.05) and no effect of treatment*decibel interaction (*F*_(2.4,17.0)_ = 7.31e-1, *p* > 0.05). In dominants, there was a significant main effect of decibel (*F*_(1.9,14.8)_ = 1.27e+2, *p* = 6.62e-10), but no effect of treatment (*F*_(1,8)_ = 1.44, *p* > 0.05) and no effect of treatment*decibel interaction (*F*_(2.0,16.0)_ = 1.58, *p* > 0.05). In subordinates, there were significant main effects of treatment (*F*_(1,8)_ = 1.18e+1, *p* = 8.92e-3), decibel (*F*_(2.3,18.1)_ = 7.53e+1, *p* = 8.60e-10), and treatment*decibel interaction (*F*_(3.0,23.6)_ = 3.41, *p* = 3.44e-2). Bicuculline significantly decreased the startle response for subordinate animals. In particular, *post hoc* test showed that there was a significant difference at 85 dB (*t*_(8)_ = 4.60, *p* = 1.75e-3). Asterisks denote statistical difference between control and experimental condition at the specified dB level: *p < 0.05, **p < 0.005; paired sample *t* test. ***D–F***, One-minute recording of far field-potentials of spontaneous swimming activity before (control) and after bicuculline injections for communal, dominants, and subordinates, respectively, along with respective average swimming frequency for all animals tested (horizontal dashed lines set arbitrarily to compare swim frequencies across experimental conditions). ***G***, Box and whiskers plots of the average number of swim bursts per 1 min for each social phenotype. Box plot parameters are defined in Figure 1*G*. We performed the repeated measures of ANOVA (within-subject factor as treatment). In communals and subordinates, there were no effects of treatment (bicuculline; *F*_(1,7)_ = 2.56, *p* > 0.05 for communals; *F*_(1,8)_ = 1.45, *p* > 0.05 for subordinates). In dominants, there was a significant effect of treatment (bicuculline; *F*_(1,8)_ = 5.49, *p* = 4.72e-2).

The escape and swim circuits receive descending excitatory dopaminergic and inhibitory inputs that modulate motor activities (Oda et al., 1995; Hatta et al., 2001; Mu et al., 2012; Medan and Preuss, 2014; McPherson et al., 2016; Tabor et al., 2018). One possible source of inhibitory inputs to M-cells is a GABAergic input. To examine the contribution of GABA in regulating the startle response, we blocked GABA_A_ receptor with systemic injections of bicuculline. We found that startle escape response was not affected in both communal and dominant animals, but it decreased significantly in subordinates (Fig. 3*A–C*). Although startle response was not affected in communals and dominants, swimming frequency declined significantly in dominants with no observable change in communals and subordinates (Fig. 3*D–G*). The results suggest that GABAergic regulation of the startle response is socially mediated, and the decrease of startle sensitivity in subordinates points to an unidentified pathway that suppresses M-cell excitability when GABAergic input is blocked.

### Neurocomputational model of the M-cell circuit

The decrease of escape sensitivity in subordinates (Fig. 3*C*) and decrease of swimming frequency in dominants (Fig. 3*G*) following bicuculline application suggest that GABA regulates the escape and swim circuits indirectly via yet unidentified inhibitory input. We postulated that glycinergic modulation is likely involved given the fact that glycine is known to regulate spinal motor circuits in zebrafish (Liao and Fetcho, 2008; Moly and Hatta, 2011). These observations led us to the hypothesis that social experience reconfigures the synergistic interactions of the neuromodulatory pathways (GABA, glycine, and DA systems) to rebalance the excitability of the escape and swim circuits in a socially dependent manner.

**Figure 4.**
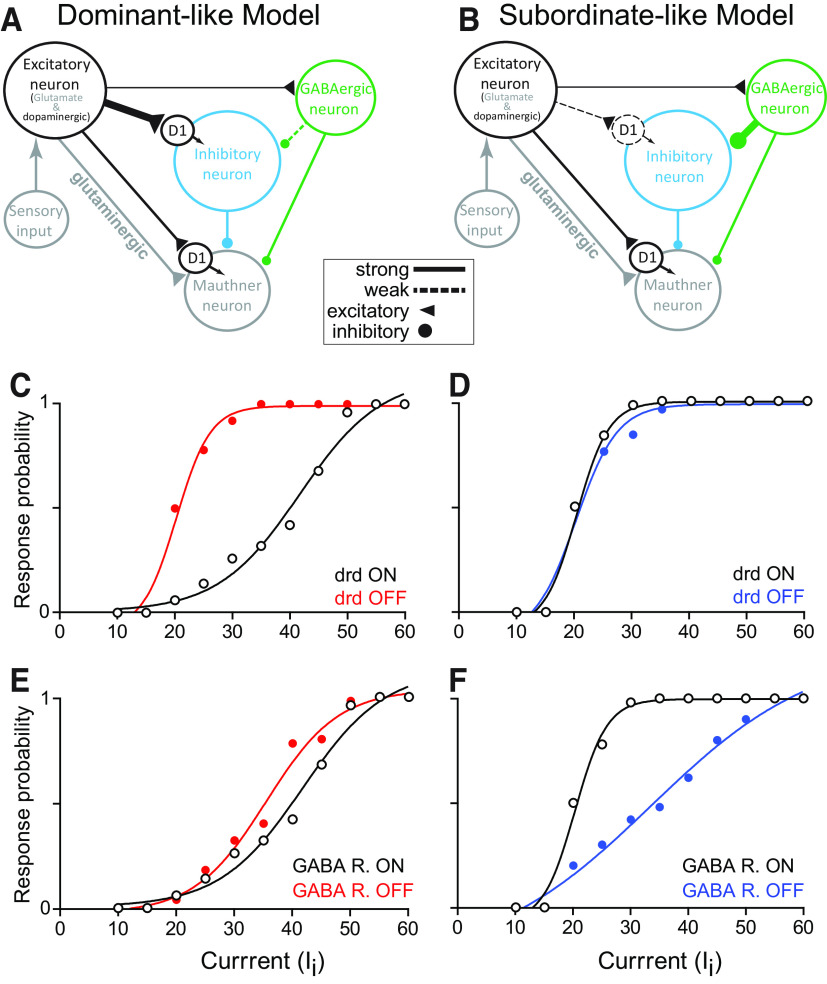
Neurocomputational model. Schematic of dominant-like (***A***) and subordinate-like (***B***) neurocomputational models. Thin solid lines, thick solid lines, and thick dashed lines represent regular inputs, strong inputs, and weak inputs, respectively. Dominant-like model was simulated with $D_{1GL}=0.65,g_{\text{GA}\to \text{GL}}=0.2$ for the WT control while subordinate-like model was simulated with $D_{1GL}=0.25,g_{\text{GA}\to \text{GL}}=0.4$ for WT control. ***C***, ***D***, Simulated response of dominant-like (***C***) and subordinate-like (***D***) models of escape circuit to positive current injections in the sensory input with/without (ON/OFF) Drd1, thus, mimicking Drd1 antagonism ( $D_{1M}$ = 0, $D_{1GL}$= 0). ***E***, ***F***, simulated response of dominant-like (***E***) and subordinate-like (***F***) models of escape circuit to positive current injections in the sensory input with/without (ON/OFF) GABAergic receptor, thus, mimicking GABAergic receptor antagonism ( $g_{\text{GA}\to \text{M}}=0$ and $g_{\text{GA}\to \text{GL}}=0$). We also used $\beta_{GL}$= 0.024 for dominant-like model and $\beta_{GL}$= 0.0072 with subordinate-like model.

To test this hypothesis, we built a neurocomputational model of the M-cell escape circuit based on the following predictions. First, DA, GABA, and glycine are socially regulated to modulate spinal motor circuits. Second, Drd1b is expressed by both the M-cell and glycinergic neurons but not by GABAergic neurons because blockage of GABAergic input exhibits the opposite startle escape responses to blockage of Drd1b (Figs. 2*B*,*C*, 3*B*,*C*). Third, GABAergic neurons regulate the escape circuit indirectly via glycinergic neurons because blockage of GABA_A_ receptor decreased the startle escape responses in subordinates (Fig. 3*C*). Within our model, we simulated the relative interactions of the three neuromodulatory inputs in our simplified neural network (Fig. 4*A*,*B*; for details, see Materials and Methods). The model is composed of conductance-based modified Morris–Lecar model neurons and simulates the M-cell and the main neuromodulatory elements known to control its excitability. The elements consist of excitatory (glutamatergic, dopaminergic) and inhibitory (GABAergic, glycinergic) neurons. We found that the model can account for the observed social status differences in circuits’ dynamics.

First, we examined how modulation of specific network properties (synaptic strengths and activation of dopamine receptors) may account for the observed differences in the shift in circuit dynamics in the two social phenotypes. We predicted that dopaminergic modulation of M-cell excitability is mediated indirectly via an inhibitory pathway, and this pathway is stronger in dominants compared with subordinates (Fig. 4*A*,*B*). This assumption was based on the result that blockage of the Drd1b receptor, which is typically associated with excitatory second messenger pathways enhanced startle probability in dominants, and *drd1b*^(−/−)^ animals displayed a heightened escape probability (Surmeier et al., 2007). This indicates a removal of a strong inhibitory drive as observed in dominants that limits M-cell excitability via activation of the Drd1b (Fig. 2*B*). To test this hypothesis, we controlled the probability of activation of the Drd1 receptor on both the M-cell and glycinergic cell ( $D_{1M}$ and $D_{1GL}$; see Fig. 4*A*,*B* and below for details). We applied repeated depolarizing current pulses to the model excitatory cell (1-s interstimulus interval for 2 ms in duration) to determine the dynamic range of the model M-cell excitability in dominant-like and subordinate-like models. To simulate blockage of the Drd1, we let $D_{1M}=0$ on the M-cell and $D_{1GL}=0$ on the glycinergic neuron. Note that the higher $D_{1GL}$, the lower response curve because of the strong glycinergic→M-cell pathway. Different effects with Drd1 antagonist in different animal groups imply that the values of $D_{1GL}$ depend on the social status. Here, $D_{1GL}$ is lower in subordinates compared with dominants. Our simulations showed that blockage of the Drd1 by turning-OFF its contribution led to a significant increase in M-cell excitability in the dominant-like model with no observable change in M-cell activity in the subordinate-like model consistent with the results observed empirically (compare Figs. 2B,*C*, and 4*C*,*D*).

To examine the contribution of GABAergic input in modulating the startle response, we also analyzed whether GABAergic regulation of M-cell excitability is mediated indirectly by differentially modulating glycinergic release in dominant and subordinate animals (Fig. 4*A*,*B*). In the dominant-like model, we found that the inhibition of GABAergic neuron to glycinergic neuron is weak so that removal of GABA would not alter the overall startle response curve (Fig. 4*E*). However, in the subordinate-like model, the GABAergic-glycinergic connection is strong, and when blocked, glycinergic neurons are dis-inhibited allowing glycine to inhibit the M-cell (Fig. 4*F*). Blockage of the GABAergic receptor was simulated by setting GABAergic synaptic strength onto glycinergic neuron and the M-cell to zero ( $g_{GA\to GL}=0,g_{GA\to M}=0)$ and also by decreasing the decay time constant β of the synaptic variable for glycinergic neuron as was shown previously in the auditory system in rats (Lu et al., 2008). Here, $g_{A\to B}$ was the maximal synaptic conductance from A to B (for details, see Materials and Methods). The decrease of decay time constant β of the synaptic variable prolonged the inhibition from the glycinergic neuron onto the M-cell, which resulted in a decrease of startle escape response curve in subordinate-like model (Fig. 4*F*) as was observed empirically (Fig. 3*C*). These results support the notion that dopaminergic and GABAergic inputs indirectly and concurrently regulate M-cell excitability by modulating glycinergic inputs.

### Glycinergic modulation of M-cell excitability is socially regulated

**Figure 5.**
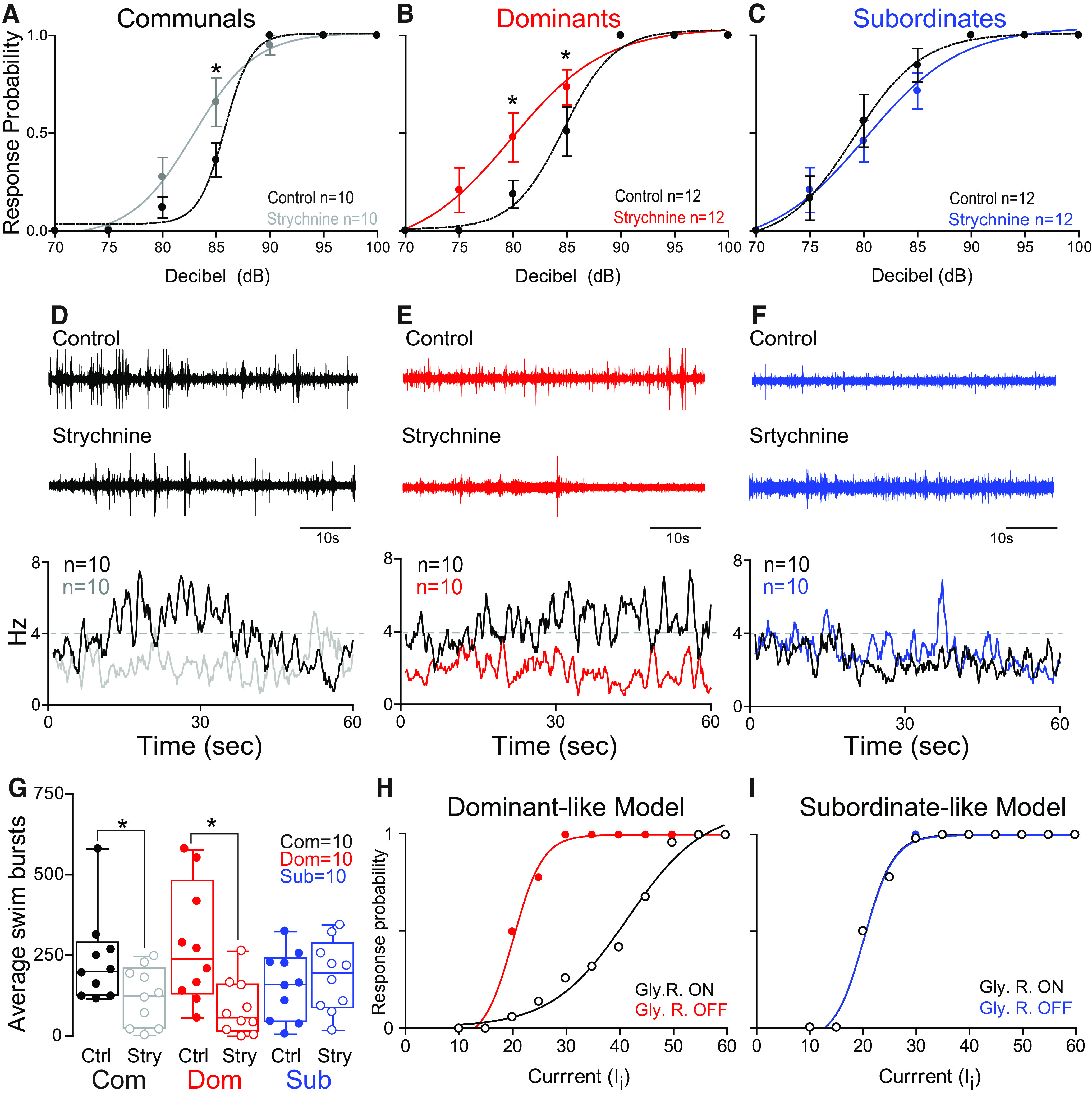
Glycinergic modulation of the escape and swim circuits is socially regulated. ***A–C***, Probability of startle escape response before (control) and after Strychnine injections for communals, dominants, and subordinates, respectively. Asterisks denote statistical difference between control and experimental condition at the specified decibel level. We performed repeated measures of ANOVA (within-subject factors as treatment and decibel) followed by one-sample two-sided *t* test for the *post hoc* test at each decibel. In communals, there was a marginal main effect of treatment (*F*_(1,9)_ = 3.71, *p* = 8.62e-2), but there were significant main effect of decibel (*F*_(1.5,13.3)_ = 1.53e+2, *p* = 8.62e-2) and significant effect of treatment*decibel interaction (*F*_(2.2,19.7)_ = 3.71, *p* = 3.96e-2). Strychnine marginally increased startle response for communal animals. In particular, *post hoc* test showed that there was a significant difference of the startle responses at 85 dB (*t*_(9)_ = 2.51, *p* = 3.32e-2). In dominants, there were significant main effect of treatment (*F*_(1,11)_ = 1.44e+1, *p* = 2.96e-3), decibel (*F*_(1.9,20.4)_ = 7.72e+1, *p* = 5.00e-10), and significant effect of treatment*decibel interaction (*F*_(2.4,25.9)_ = 4.04, *p* = 2.45e-2). Strychnine significantly increased the startle response for dominant animals. In particular, *post hoc* test showed that there was a marginal difference of the startle responses at 75 dB (*t*_(11)_ = 1.82, *p* = 9.60e-2), but there were significant differences at 80 dB (*t*_(11)_ = 2.65, *p* = 2.24e-2) and 85 dB (*t*_(11)_ = 2.95, *p* = 1.33e-2). In subordinates, there was a significant main effect of decibel (*F*_(2.0,21.7)_ = 5.07e+1, *p* = 6.99e-9), but no effect of treatment (*F*_(1,11)_ = 1.60, *p* > 0.05) and no effect of treatment*decibel interaction (*F*_(2.3,25.8)_ = 1.28, *p* > 0.05). ***D–F***, One-minute recording of far field-potentials of spontaneous swimming activity before (control) and after strychnine injections for communal, dominants, and subordinates, respectively, along with respective average swimming frequency for all animals tested (horizontal dashed lines set arbitrarily to compare swim frequencies across experimental conditions). ***G***, Box and whiskers plots of the average number of swim bursts per 1 min for each social phenotype. Box plot parameters are defined in Figure 1*G*. We performed the repeated measures of ANOVA (within-subject factor as treatment). In communals (*n* = 10) and dominants (*n* = 10), there were significant effects of treatment (strychnine; *F*_(1,9)_ = 6.06, *p* = 3.61e-2 for communals; *F*_(1,9)_ = 1.06e+1, *p* = 9.88e-3 for dominants). In subordinates (*n* = 10), there was no effect of treatment (strychnine; *F*_(1,9)_ = 8.76e-3, *p* > 0.05). ***H***, ***I***, Simulated response of dominant-like (***H***) and subordinate-like (***I***) models of escape circuit to positive current injections in the sensory input with/without (ON/OFF) GlyR, thus, mimicking GlyR antagonism ( $g_{\text{GL}\to \text{M}}=0$).

To investigate the model’s prediction that the glycinergic input is weaker in subordinates compared with dominants, we blocked glycinergic transmission with systemic injections of strychnine, a glycine receptors (GlyR)-specific antagonist. We found that strychnine significantly increased startle sensitivity in dominants and marginally increased startle response in communals but not in subordinates (Fig. 5*A–C*). These results suggest that glycinergic modulation of M-cell excitability is weaker in subordinates compared with dominants because of the strong GABAergic inhibition onto glycinergic neurons as was demonstrated when GABA_A_ receptor was blocked that led to a strong reduction in M-cell excitability (Fig. 3*C*). While the probability of the startle response was increased in communals and dominants in the presence of strychnine, swimming frequency was significantly reduced in both communals and dominants with no observable change in subordinates (Fig. 5*D–G*).

We simulated the switch in M-cell excitability in our neurocomputational model by blocking GlyR; thus, reducing the synaptic strength between the glycinergic neuron and the M-cell to zero ( $g_{GL\to M}=0)$. The dominant-like model simulated a significant increase in M-cell excitability when the GlyR was turned-OFF while the subordinate-like model showed little change as was observed empirically (Fig. 5*H*,*I*). Collectively, our results show that glycinergic modulation of M-cell excitability is socially mediated in that the synaptic strength of glycinergic input onto the M-cell is weaker in subordinates, compared with dominants and communals, and that glycinergic, dopaminergic, and GABAergic inputs work synergistically to differentially regulate M-cell excitability in dominant and subordinate zebrafish.

### Status-dependent expression of Drd1b in glycinergic neurons

**Figure 6.**
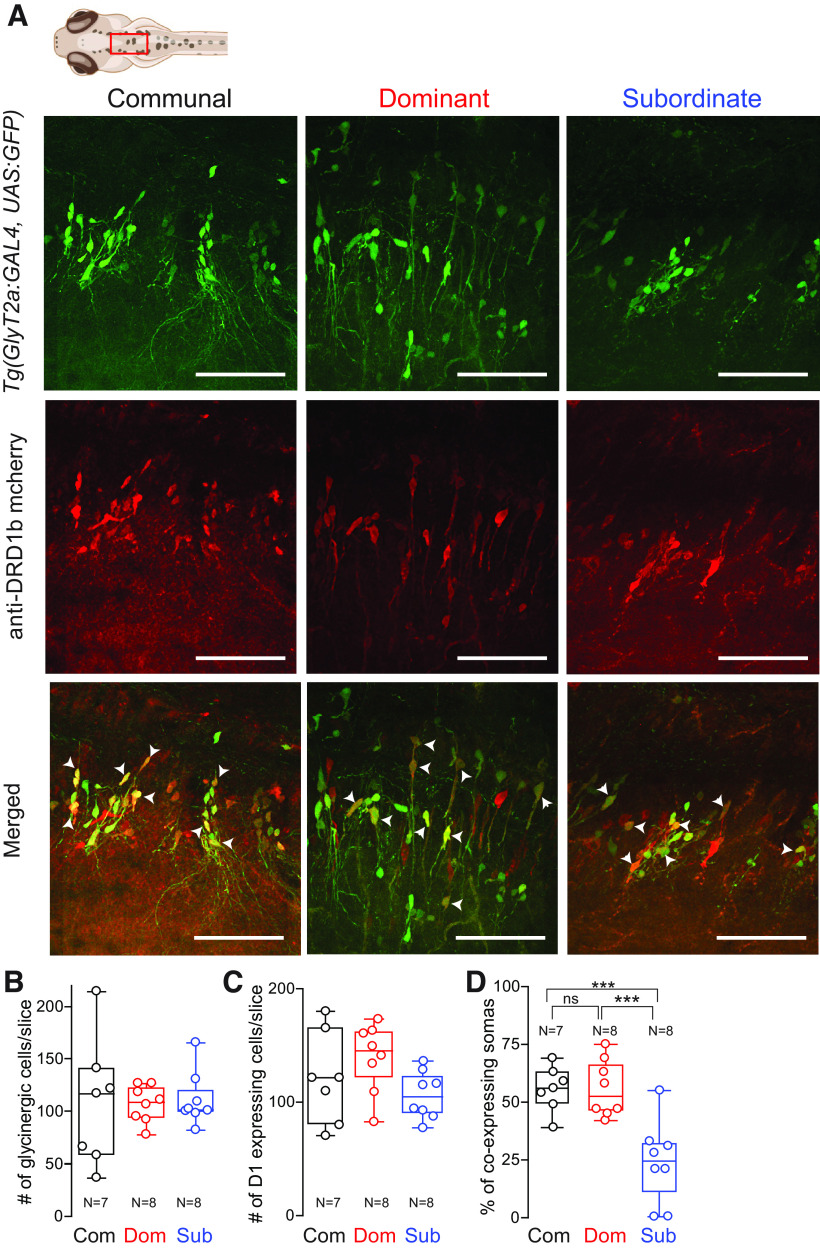
Status-dependent expression of Drd1b in hindbrain glycinergic neurons. ***A***, Representative confocal projections of optical sections of communal, dominant, and subordinate animals. Top row, Images of glycine neurons. Second row, Drd1b staining. Third row, Glycine and Drd1b merged. Arrowheads point to co-expressing cells. Projections, Dashed lines denote midline; anterior is to the left. ***B–D***, Comparing the numbers of glycinergic cells per brain slice (***B***), the numbers of D1 expressing cells per brain slice (***C***), and the percentage of co-expression of Drd1b and glycinergic neurons (***D***) among communal (*n* = 7), dominant (*n* = 8), and subordinate (*n* = 8) animals. We performed one-way ANOVA (between-subject factor as group) followed by Tukey’s HSD *post hoc* test for the group comparisons. ***B***, There was no effect of group (*F*_(2,20)_ = 2.81e-1, *p* > 0.05). ***C***, There was no effect of group (*F*_(2,20)_ = 2.27, *p* > 0.05). ***D***, There was a significant main effect of group (*F*_(2,20)_ = 1.38e+1, *p* = 1.73e-4). The *post hoc* tests showed a significant decrease in co-expression for subordinates compared with communals (*p* = 7.27e-4) and dominants (*p* = 4.79e-4). Scale bar = 50 μm.

Our results suggest dopaminergic regulation of M-cell excitability is mediated indirectly via glycinergic input, and the DA→glycinergic input in subordinate animals is weaker compared with dominants. Given the differences of *drd1b* gene expression between the two social phenotype (Fig. 1*C*,*D*), we postulated that hindbrain glycinergic neurons would show lower expression of Drd1b in subordinates animals compared with dominants. If so, then it would explain why subordinates have enhanced startle sensitivity. To test this hypothesis, we examined Drd1b expression using the transgenic line [Tg*(GlyT2a:GFP*)] that expresses GFP specifically in glycinergic neurons (McLean et al., 2007). Using confocal imaging along with Imaris digital colocalization analysis (for details, see Materials and Methods), we quantified the total number of glycinergic neurons and cells that express Drd1b receptor (Fig. 6). We focused on a subpopulation of hindbrain glycinergic neurons whose commissural axonal projections synapse onto and provide feed-forward inhibitory regulation of M-cell excitability (Faber et al., 1989; Eaton et al., 2001; Moly and Hatta, 2011). We found that the total number of hindbrain glycinergic neurons did not differ among the three social groups (Fig. 6*B*). Although, the overall number of Drd1b expressing cells showed a modest decrease in subordinates, the differences among animal groups were insignificant (Fig. 6*C*). However, Drd1b expression specifically within the glycinergic neurons decreased significantly in subordinates compared with dominants and communal fish (Fig. 6*D*; Movies 1, 2, 3). This result is consistent with the neurocomputational model in that dopaminergic modulation of M-cell excitability is mediated, in part, indirectly via modulation of glycinergic neurons whose expression of the Drd1b is socially regulated.

Movie 1. 3D digital illustration of hindbrain glycinergic nucleus expressing the Drd1b receptor in communal zebrafish brain. 10.1523/ENEURO.0311-23.2023.video.1

Movie 2. 3D digital illustration of hindbrain glycinergic nucleus expressing the Drd1b receptor in dominant zebrafish brain. 10.1523/ENEURO.0311-23.2023.video.2

Movie 3. 3D digital illustration of hindbrain glycinergic nucleus expressing the Drd1b receptor in subordinate zebrafish brain. 10.1523/ENEURO.0311-23.2023.video.3

## Discussion

Here, we examined how social experience shapes the synergistic interactions of multiple neuromodulators (DA, GABA, glycine) to regulate the activity of two mutually exclusive motor behaviors: escape and swimming. Although we limited the architecture of the neurocomputational model to the escape circuit with no elements of the swim circuit incorporated, the combined empirical and computational approach lead to three principal conclusions. First, the balance in activation of the escape and swim circuits is socially regulated by shifting the balance of excitatory and inhibitory pathways onto the escape and swim circuits. Second, the shift in network activity is synergistic. Connections are adaptively strengthened and weakened accordingly to promote the proper initiation of a specific motor program. In turn, multiple neuromodulatory networks maintain a dynamic balance to provide inputs that alter motor circuit excitability. Third, the DA signaling pathway is a key molecular pathway that underlies status-dependent excitability of the escape and swim motor circuits.

**Figure 7.**
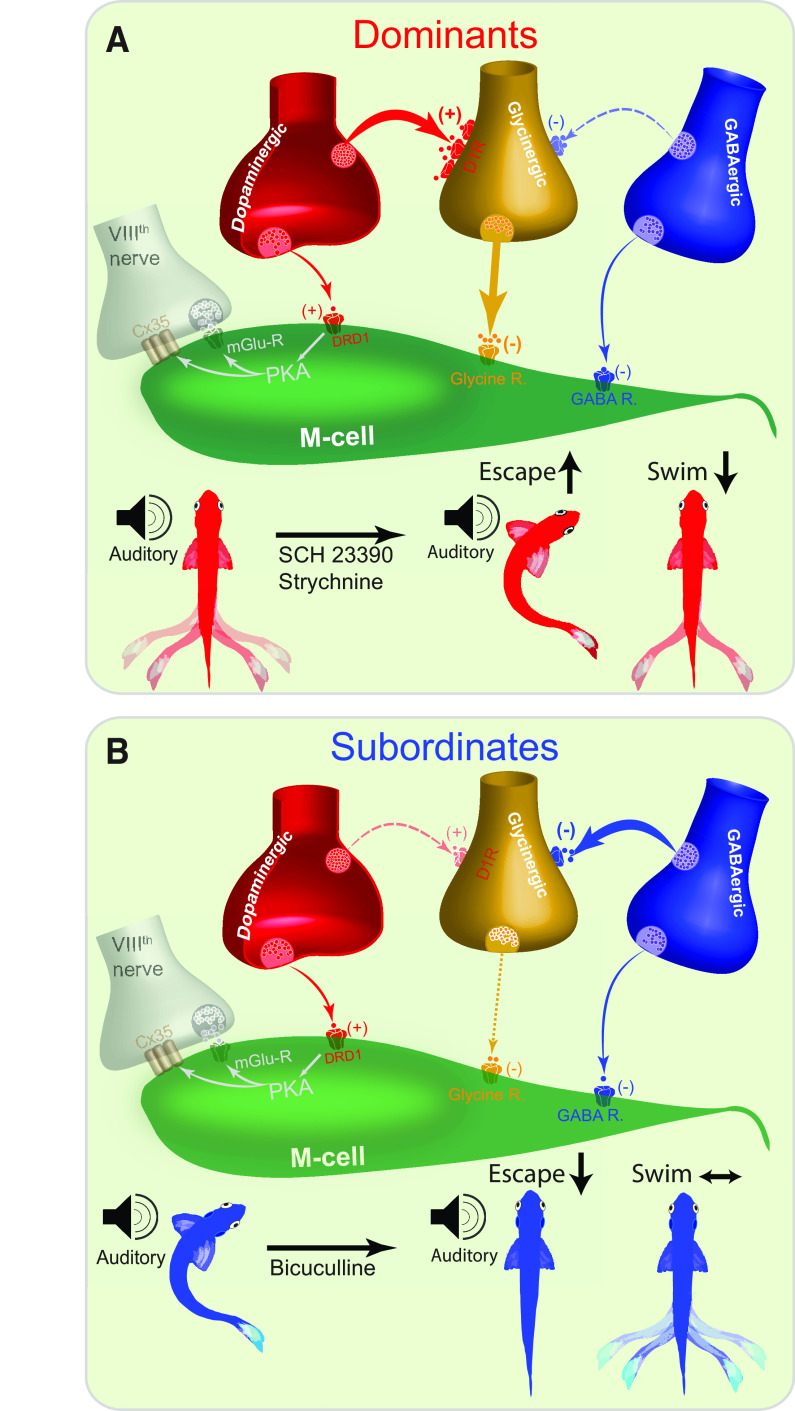
Proposed model of socially mediated shift in synaptic reconfiguration underlies synergistic modulation of the motor circuits. ***A***, Dominants proposed model: the relatively higher Drd1b expression in glycinergic neurons strengthens the dopaminergic→glycinergic synapse, promoting glycine release, and inhibition of the M-cell. The disproportionately stronger dopaminergic→glycinergic pathway compared with the weak GABAergic→glycinergic input culminates in the activation of the glycinergic neurons and lower excitability of the M-cell. Lower panel, Blockage of either Drd1b or GlyR facilities behavioral switch from swimming to escape. ***B***, Subordinates proposed model: the disproportionately weaker dopaminergic→glycinergic pathway because of decreased Drd1b expression in the glycinergic neurons culminates in the reduced activity of the glycinergic neurons and enhanced excitability of the M-cell. Lower panel, Blockage of either Drd1b or GlyR has no effect on escape or swimming activity. However, blockage of GABAergic input promotes dominant like locomotor behavior that promotes swimming over escape. Proposed strong pathways are illustrated as solid colors, while weak pathways are illustrated as faded colors. (+) denotes excitatory synapse, (−) denotes inhibitory synapse.

Evidence from this study supports our proposed model of how social experience regulates the excitability of the escape and swim circuits (Fig. 7). Our results suggest the presence of synaptic connections between the dopaminergic and glycinergic neurons (Yao et al., 2016) and GABAergic and glycinergic neurons. In dominant animals, the synaptic pathway of the dopaminergic→glycinergic→M-cell is strong, while the GABAergic→glycinergic pathway is weak (Fig. 7*A*). This conclusion is supported by the evidence that glycinergic neurons show higher Drd1b expression compared with subordinates, and blockage of either Drd1b or GlyR shifts the balance of circuit activity from swimming to escape (Figs. 2, 5, 6). Conversely, in subordinates the synaptic drive of the dopaminergic→glycinergic→M-cell is weak because of decreased Drd1b expression in glycinergic neurons, allowing for a stronger inhibitory drive from the GABAergic→ glycinergic pathway (Fig. 7*B*). This rebalance decreases glycine’s inhibition of the M-cells and leads to an enhancement of M-cell excitability. This is supported by the result that the probability of escape response in subordinates was reduced significantly only when GABA_A_ receptors were blocked (Fig. 3*C*). This shift in excitability illustrated in the strengthening of the inhibitory (GABA→glycine) pathways culminates with a net inhibition of the glycinergic neurons and an increase in M-cell excitability (Fig. 7*B*).

Our results show that status-dependent differences in Drd1b expression in glycinergic neurons drive the DA→glycinergic synaptic plasticity. Indeed, hindbrain glycinergic neurons showed higher Drd1b expression in dominants compared with subordinates (Fig. 6*A*,*B*). The higher expression of Drd1b in dominants in the glycinergic neurons is likely to increase dopaminergic→glycinergic synaptic strength and DA’s ability to activate the glycinergic neurons, thus, reducing M-cell excitability. In subordinates, reduced expression of the Drd1b in hindbrain glycinergic neurons is likely to exert the opposite effect by weakening the synaptic drive of the dopaminergic→glycinergic neurons mimicking dominants response phenotype (Figs. 2*B*, 7*B*). This DA→glycine connection has been previously established, where a specific glycinergic cluster in the hindbrain has been found to make synaptic contacts on the soma of the M-cells (Seitanidou et al., 1988). Although we do not have direct evidence, our results suggest that GABAergic neurons do not express the Drd1b at a significant level. If the Drd1b was sufficiently expressed by GABAergic neurons, then antagonizing Drd1b function would have weakened the GABA→glycine pathway, removing the glycinergic neurons from inhibition, which would have increased glycine release leading to a decrease in M-cell excitability. However, we observed the opposite effect supporting the notion that GABAergic neurons do not express the Drd1b receptor (Figs. 2*B*, 7).

As with many teleost fish, zebrafish rely on visual and olfactory cues to transmit social information to decision-making nuclei (Clements et al., 2018; Nunes et al., 2020; Pinho et al., 2020). Recent evidence has shown that the habenula is an important nucleus in relaying social information to motor circuits (Hikosaka, 2010; Chou et al., 2016). Specifically, the habenula integrates aversive cues processed by the limbic system and basal ganglia and relay that information to hindbrain circuits to adapt motor responses according to environmental conditions (Hikosaka, 2010). The lateral habenula is of particular interest because of its control over the dopaminergic and serotonergic systems both of which are implicated in motor control, aggression, anxiety, and depression (Proulx et al., 2014). Importantly, prior studies have shown that dopaminergic nuclei in the mid and hindbrain modulate spinal motor and premotor circuits including direct innervation of the M-cells (McLean and Fetcho, 2004; Kastenhuber et al., 2010; Tay et al., 2011; Mu et al., 2012; Jianhua et al., 2017; Haehnel-Taguchi et al., 2018; Barrios et al., 2020). Recent evidence has shown that a specific dopaminergic cluster of the descending diencephalospinal system whose projections innervate the spinal cord are socially regulated, whereby the number of DA cells increases in socially dominant zebrafish compared with their subordinate counterparts (Heagy et al., 2022). Although the functional consequence of this morphologic plasticity on motor activity remains unknown, the results suggest that the diverse forms of plasticity that impinge on M-cell excitability are not limited to synaptic and biochemical plasticity, but it is likely broader in scope and includes neurogenesis and morphologic reconfiguration of descending inputs that modulate spinal motor circuits. This is consistent with prior studies in other organisms highlighting the impact of social experience on brain structure and function as an adaptation to new social conditions (Hofmann and Fernald, 2000; Whitaker et al., 2011; O’Connell and Hofmann, 2012; Weitekamp et al., 2017; Inada et al., 2022; Lee et al., 2022; Liu et al., 2022).

Additional mechanisms underlying status-dependent plasticity of the escape circuit could also be at play. One possibility that remains unexplored is whether the changes in M-cell excitability is because of status-dependent expression of GlyR in the M-cells. Behavioral desensitization of the startle escape response was shown to be mediated by potentiation of glycinergic inputs onto the M-cells driven by increased synaptic clustering of GlyR on the M-cell somas (Ogino et al., 2019). Although speculative, it is likely that experience-driven somatic clustering of GlyR works congruently with presynaptic changes in Drd1b expression in the glycinergic neurons to regulate M-cell excitability as social dominance matures. For instance, if subordinates have decreased glycine activation because of decreased DA signaling, this may induce somatic GlyR clustering; thus, removing the M-cell from inhibition. Conversely, enhanced dopaminergic input onto the glycinergic neurons may indirectly lead to an increase in GlyR synaptic clustering because of increased glycine release. Future experiments examining M-cell GlyR expression in dominant and subordinate animals will provide further insight of how social factors influence this circuit. Finally, our results show status-dependent differences in DAT expression, which may play a role in regulating the differential supply of dopamine onto postsynaptic targets. Indeed, our results showed that supplementing exogenous dopamine through (L-DOPA injections) reverses social status-dependent differences in startle sensitivity: dominants’ startle sensitivity increases during L-DOPA, while startle decreases in subordinates during L-DOPA administration. Although the observed effects were statistically insignificant, the changes reduced the differences in startle sensitivity between dominants and subordinates (compare Fig. 2*H*, L-DOPA-injected DOM vs SUB curves). This result in conjunction with status mediated differences in Dat expression and Drd1b regulation suggests that social dominance is likely inducing broad effects on the startle escape circuit and the higher brain decision-making nuclei that modulate startle escape sensitivity as an adaptation to changes in social rank.

The ecological implications of the status-dependent differences in motor activity remain unknown. Reduction in swimming in subordinates is an important behavioral strategy because it minimizes the frequency of interactions with the dominant fish while prioritizing escape with an increase in startle escape sensitivity. Conversely, the increase in swimming in dominants may be because of an increase in territorial displays as dominance solidifies between opponents. Indeed, prior work investigating spatial distribution of zebrafish during social interactions showed clear differences in swimming activity whereby subordinates reduce their swimming and occupy the bottom corner of the aquarium to avoid interacting with the dominant counterparts, thus, avoiding persistent aggression. Conversely, dominant fish increase their swimming frequency during social interactions as part of their territorial display (Miller et al., 2017). These behavioral adaptations to changes in social conditions are not limited to zebrafish. Prior work investigating the ecological implications of social dominance on startle escape behavior in Cichlid (*Astatotilapia burtoni*) showed that males differ in visual conspicuousness based on social status: dominants are brighter in coloration compared with subordinates (Whitaker et al., 2021). Because of their visual conspicuousness, dominants are at a higher risk of predation. They compensate for this potential risk by enhancing their probability of executing a startle escape response compared with their less conspicuous counterparts.

In conclusion, our findings show that social regulation of motor behavior is mediated by synergistic interactions of multiple neuromodulatory signaling pathways that shift motor circuit dynamics to optimize motor selection according to social rank. This serves as an adaptive behavioral strategy as animals rise and fall in social dominance and is likely an evolutionarily conserved cellular mechanism employed by other social species.

## Synthesis

Reviewing Editor: David Schoppik, New York University Decisions are customarily a result of the Reviewing Editor and the peer reviewers coming together and discussing their recommendations until a consensus is reached. When revisions are invited, a fact-based synthesis statement explaining their decision and outlining what is needed to prepare a revision will be listed below. The following reviewer(s) agreed to reveal their identity: Hans Hofmann, Jens Herberholz.

1) What was the rationale for using the Boltzmann sigmoidal equation to approximate the startle response vs. stimulus intensity data (lines 130ff)? We understand that this model is commonly used in this context. But the reader would benefit from a brief justification.

2) The Discussion does a good job of placing the results within the larger context of the literature, and the authors are justified in emphasizing the role of the described plasticity in social dominance hierarchies. The authors may also want to consider whether and how other aspects of life history may differ between dominant and subordinate individuals and consequently favor differences in motor activity and startle behavior, including ecological attributes such as differential predation risk. Specifically, c.f. cichlid fish, see Whitaker et al. (Hydrobiologia 848:3667, 2021;https://doi.org/10.1007/s10750-020-04475-9).

3) qPCR results are normalized to isolated fish while all other experiments compare Subs and Doms with communally housed conspecifics (Coms).

a. What is the reason for changing the “control” from isolates to communals in later parts of the study?

b. What does a communal animal represent? There isn’t any explanation other than housing was done in a tank with approx. 20 animals. Wouldn’t animals in communal tanks form social hierarchies with Doms and Subs being present among them? If Coms were chosen randomly from the tank, what are the probabilities to select a Dom or Sub? The results seem to suggest that Coms are more similar to Doms, but is this expected? It’s not discussed in the manuscript. Maybe finding a few Doms and mostly Subs in a communal tank would be more likely? This point needs further explanation/discussion.

4) Aside from the dopamine receptor, qPCR also revealed a major difference between Subs and Doms in the expression of the dopamine transporter.

a. What is the significance of Doms having more transporter (and presumably higher reuptake)? Unless we missed it, there wasn’t any further investigation, neither experimentally by pharmacology or other methods, nor was it included in the circuit model(s), or mentioned in the discussion section. This should be addressed.

5) Most of the hypotheses, data, models, and graphs are focused on the Mauthner circuit rather than the swim circuit while the abstract suggests that the study investigates the interplay between both circuits and behaviors.

a. Unless there is a direct relationship between M-cell excitability and swimming, we are not sure how much the swim data contributes to the overall story. It mainly comes across as an interesting observation that is weakly integrated with the detailed circuit mechanisms that shape M-cell modulation. We suggest adding additional explanation for (possible) interconnections between Mauthner escape and swimming, or maybe some other ways to better integrate the current results across both motor patterns.

b. The behavioral relevance of changes in swimming activity is not explained. We can understand why Subs might lower M-cell escape threshold, but it’s less clear why Subs would swim less than Doms (or Doms more than Subs). Maybe previous publications have addressed this point, but it’s still important to mention it here.

Specific comments:

1. Fig. 1 A,B: Related to our comments above, maybe a simple schematic illustrating both circuits (M-cell, Swim), including the outputs to the motor system and any interactions, would be useful? Figure 1A: Consider labeling the dendrites on the Mauthner cell cartoon for clarity, Figure 1C,D: Consider displaying the data as box plots with overlaid data points, similar to subsequent figures (e.g., 1G). This gives the reader a better picture of the data.

2. Since groups of mixed sex were used, was an effect of sex measured/found in any of the data?

3. How was behavioral data scored to determine Doms/Subs? While this may follow an established protocol (Miller et al.), a brief explanation would be useful.

4. A rationale for excluding long-latency escapes (>15 ms) might be desirable. One might assume that inhibition of the M-cell (via the proposed mechanisms) will lead to more non-Mauthner escapes?

5. Was swimming activity ever video-recorded? Was the electrical signal from the water-electrodes matched with video at some point (e.g., in a previous study/publication) to confirm its reliability?

6. Did the statistical analysis consider relevant assumptions for ANOVA - e.g., no outliers, normal distribution, equal variance across groups? Please provide statistical justification for the analysis

7. The same concentration (20 mM) was used for all drugs (and adjusted for weight). How was this decided? Is there existing literature supporting this specific concentration? Did the injection ever cause any “unusual” behaviors? For example, bicuculline is commonly used to induce seizures in mammals.

8. The L-DOPA results are not significant and may not be all that relevant; they are, however, somewhat confusing to me. Wouldn’t you expect the opposite finding (based on the results with the DR1 antagonist)?

9. The reduced number of DR1 receptors on glycinergic neurons of Subs is an interesting finding. However, the circuit diagrams, Fig. 4 & 7 (and the graphical abstract), suggest that the higher excitability of the M-cell is largely determined by increased GABAergic inhibition of these neurons (i.e., inhibition of inhibition). This is further supported by the bicuculline experiment. Thus, using an antibody to label GABA receptors would have been informative. Maybe this wasn’t feasible, or there is some other explanation?

Line-by-line

- Lines 103ff: How were the sound stimuli generated (loudspeaker model, placement, etc.)?

- Lines 153ff: The authors should describe from which tissue RNA was extracted, and how this tissue was dissected and processed. This information is provided for the western blot and IHC sections.
